# microRNA profiles in urine by next-generation sequencing can stratify bladder cancer subtypes

**DOI:** 10.18632/oncotarget.25057

**Published:** 2018-04-17

**Authors:** Barbara Pardini, Francesca Cordero, Alessio Naccarati, Clara Viberti, Giovanni Birolo, Marco Oderda, Cornelia Di Gaetano, Maddalena Arigoni, Federica Martina, Raffaele A. Calogero, Carlotta Sacerdote, Paolo Gontero, Paolo Vineis, Giuseppe Matullo

**Affiliations:** ^1^ Italian Institute for Genomic Medicine, Turin, Italy; ^2^ Department of Medical Sciences, University of Turin, Turin, Italy; ^3^ Department of Computer Science, University of Turin, Turin, Italy; ^4^ Department of Surgical Sciences, University of Turin and Città della Salute e della Scienza, Turin, Italy; ^5^ Molecular Biotechnology Center, Department of Biotechnology and Health Sciences, University of Turin, Turin, Italy; ^6^ Center for Cancer Prevention, CPO-Piemonte, Turin, Italy; ^7^ MRC-HPA Centre for Environment and Health, School of Public Health, Imperial College London, London, United Kingdom

**Keywords:** bladder cancer, microRNA profiling, urine biomarkers, next-generation sequencing, liquid biopsy

## Abstract

Bladder cancer (BC) is the most frequent malignancy of the urinary tract with a high incidence in men and smokers. Currently, there are no non-invasive markers useful for BC diagnosis and subtypes classification that could overcome invasive procedures such as cystoscopy. Dysregulated miRNA profiles have been associated with numerous cancers, including BC. Cell-free miRNAs are abundantly present in a variety of biofluids including urine and make them promising candidates in cancer biomarker discovery.

In the present study, the identification of miRNA fingerprints associated with different BC status was performed by next-generation sequencing on urine samples from 66 BC and 48 controls. Three signatures based on dysregulated miRNAs have been identified by regression models, assessing the power to discriminate different BC subtypes. Altered miRNAs according to invasiveness and grade were validated by qPCR on 112 cases and 65 controls (among which 46 cases and 16 controls were an independent group of subjects while the rest were replica samples).

The area under the curve (AUC) computed including three miRNAs (miR-30a-5p, let-7c-5p and miR-486-5p) altered in all BC subtypes showed a significantly increased accuracy in the discrimination of cases and controls (AUC model = 0.70; *p*-value = 0.01).

In conclusions, the non-invasive detection in urine of a selected number of miRNAs altered in different BC subtypes could lead to an accurate early diagnosis of cancer and stratification of patients.

## INTRODUCTION

Bladder cancer (BC) is among the most frequent malignancies worldwide, with an estimated 429,000 new cases in 2012 [1]. BC is a highly heterogeneous disease. The largest portion of cases (70%) is non-muscle-invasive BC (NMIBC), confined to mucosa or submucosa and with superficial, non-infiltrating lesions. The remaining subset of cases is classified as muscle-invasive BC (MIBC) [2]. There is a quite high percentage of NMIBC (50–70%) that will recur, and roughly 10–30% will progress to MIBC [3].

BC screening and early diagnosis have primary importance in improving survival and quality of life of patients. Urine cytology is currently the most commonly non-invasive test used for BC detection but is of limited value owing to its poor sensitivity, especially for low-grade lesions [4]. Cystoscopy-guided biopsy for histological evaluation can offer high diagnostic accuracy but it is invasive and inconvenient for patients. BC is also among the most expensive cancers and poses a significant economic and social challenge, as the high rate of recurrences requires continuous cystoscopic surveillance [5]. Hence, non-invasive and more sensitive molecular biomarkers are needed to improve current strategies for the detection and monitoring of this cancer, particularly in patients` biofluids [6, 7].

microRNAs (miRNAs) are a class of small non-coding RNAs that act as post-transcriptional regulators in gene expression silencing by binding to complementary messenger RNA. Deregulated miRNA profiles have been associated with numerous cancers, including BC [8]. Cell-free miRNAs are abundantly present in a variety of biofluids including plasma, saliva, and urine [9–11]. The easy accessibility of several biofluids and the remarkable stability of cell-free miRNAs make them promising candidates in cancer biomarker discovery [7].

In the last years, the research on BC has focused on urinary markers and proposed several candidates but only some were validated in independent populations [12]. Frequently, studies were based on heterogeneous groups of patients, with no attention to the subtype characterization. Moreover, no studies were conducted to profile urinary miRNAs at whole miRNome level using deep sequencing techniques [13].

In the present study, we investigated urinary miRNA profiles in association with BC and different clinicopathological subtypes by next-generation sequencing (NGS). Candidate miRNAs were validated by real-time quantitative PCR (qPCR). The most interesting miRNAs were included in a model to test their power in predicting BC.

## RESULTS

### Discovery

In total, 114 samples (from 66 BC cases and 48 controls) were used in the analyses. Among cases, ten were diagnosed MIBC while 56 were NMIBC (39 G1 + G2 and 17 G3) (Table 1).

**Table 1:** Baseline patient and tumor characteristics of the samples included in the study

		Discovery (*n*)		Validation (*n*)		Overall (*n*)	
		Cases (66)	Controls (48)	Cases (46)	Controls (16)	Cases (112)	Controls (65)
**Age**	**Mean (median)**	64.27 (65.02)	64.64 (65.60)	64.63 (66.83)	65.14 (70.25)	64.42 (65.89)	64.76 (66.53)
	**Intervals**	44.92–74.10	46.44–74.91	46.78–74.64	41.92–74.49	44.92–74.64	41.92–74.91
**Smoking**	**non smokers**	7	5	3	6	10	11
	**former smokers**	34	26	22	6	56	32
	**current smokers**	25	18	21	3	46	21
	**n.a.**	0	0	0	1	0	1
**Grade**^#^	**G1**	14		16		30	
	**G2**	25		27		52	
	**G3**	27		3		30	
**G**^#^	**High grade**	41		14		55	
	**Low grade**	25		32		57	
**Tumor Stage**	**Tis**	3		1		4	
	**Ta**	29		35		64	
	**T1**	24		8		32	
	**≥T2**	10		0		10	
	**TX**	0		2		2	
Tumor Type^*^	**NMIBC**	56		46		102	
	**MIBC**	10		0		10	
**Risk**^#*^	**1**	11		15		26	
	**2**	18		22		40	
	**3**	26		9		35	
	**MIBC**	10		0		10	
	**n.a.**	1		0		1	
**Recurrences**	**yes**	25		15		40	
	**no**	41		31		72	
**Progression**	**yes**	2		0		2	
	**no**	64		46		110	
**Status**	**Alive**	58		44		102	
	**Dead**	8		2		10	

^*^According to EAU Guidelines on Non-Muscle-invasive Urothelial Carcinoma of the Bladder: Update 2013; European urology. 2013; 64:639–653.

^#^According to Cheng L. Cancer: 2000; 88:1513–6. and Montironi R. Lopez-Beltran A. International Journal of Surgical Pathology. 2005; 13:143–53.

Details about the sample features are reported in Supplementary Table 1 and Supplementary Results. The analysis of the raw reads has led to the definition of the starting count matrix composed of 114 samples and 1822 miRNAs having at least one read in one sample (see Supplementary Material and Methods). However, in the analyses of the Discovery phase, only miRNAs having at least 20 counts considering all samples (1787 miRNAs out of 1822) were included.

In the comparison between NMIBC G1 + G2 and controls, 98 differentially expressed miRNAs were found, and 14 of them had high read abundance (from hereby called DEmiRNAs). Five miRNAs (miR-30a-5p, miR-205-5p, miR-584, let-7c and miR-7706) were associated with a Predictive Power (PP) higher than 0.7 by logistic regression analysis (PPmiRNAs) **(**Supplementary Table 2A). Following the criteria described in Supplementary Material and Methods, seven candidates as NMIBC G1 + G2 biomarkers were singled out (reported in Table 2). Plot counts are shown in Supplementary Figure 1.

**Table 2:** List of all candidate miRNAs from the discovery phase stratified for BC invasiveness that were selected for replica/validation phase

	Mean read counts	log2 Fold Change	adj *P* (FDR)	PP
Candidate miRNAs for MIBC				
^a^miR-21-5p	29223	1.42	0.01	0.85
^a^miR-106b-3p	130	2.53	0.00	0.85
^b^miR-30a-5p	19453	–1.80	0.05	0.85
^b^let-7c-5p	1497	–1.62	0.02	0.85
^b^miR-486-5p	819	3.68	0.01	<0.70
^c^miR-205-5p	376	2.97	0.00	0.9
miR-451a	1004	3.40	0.04	<0.70
miR-25-3p	588	1.99	0.02	<0.70
miR-7-1-5p	518	3.32	0.05	0.85
miR-146a-5p	420	2.49	0.04	<0.70

Candidate miRNAs for NMIBC G1 + G2				
^d^miR-30c-2-5p	2106	–0.73	0.00	<0.70
^d^miR-151a-3p	2873	0.34	0.02	<0.70
^b^miR-30a-5p	27229	–0.63	0.00	0.73
^b^let-7c-5p	2644	–0.43	0.04	0.71
^b^miR-486-5p	1868	1.94	0.02	<0.70
^c^miR-205-5p	479	1.82	0.00	0.73
let-7i-5p	5038	0.55	0.03	<0.70

Candidate miRNAs for NMIBC G3				
^a^miR-21-5p	38624	0.87	0.03	0.76
^a^miR-106b-3p	184	2.30	0.00	0.88
^b^miR-30a-5p	29232	–1.67	0.00	0.76
^b^let-7c-5p	2939	–1.17	0.03	<0.70
^b^miR-486-5p	2918	3.14	0.00	<0.70
^d^miR-30c-2-5p	2241	–1.47	0.01	<0.70
^d^miR-151a-3p	3884	0.51	0.03	<0.70
miR-10b-5p	28018	–1.88	0.00	0.85
miR-148b-3p	660	0.95	0.00	0.85
miR-183-5p	1414	1.50	0.00	<0.70
miR-185-5p	381	2.10	0.00	0.76
miR-200c-3p	9145	1.11	0.00	0.76
miR-224-5p	488	2.97	0.00	0.79
miR-4448	7834	–2.27	0.01	<0.70
miR-98-5p	459	1.01	0.01	0.79

Abbreviations: FDR false discovery rate, PP predictive power, MIBC muscle-invasive bladder cancer, NMIBC non-muscle invasive bladder cancer.

^a^miRNAs in common between NMIBC G3 and MIBC.

^b^miRNAs in common among NMIBC G1 + G2, NMIBC G3, and MIBC.

^c^miRNAs in common between NMIBC G1 + G2 and MIBC.

^d^miRNAs in common between NMIBC G1 + G2 and NMIBC G3.

In the second comparison (NMIBC G3 vs. controls), 263 DEmiRNAs and 61 PPmiRNAs were found (Supplementary Table 2B). For the Validation/Replica phase, we selected three miRNAs in common between DEmiRNAs and PPmiRNAs and 12 additional candidate miRNAs inspecting the plot counts (miR-30a-5p, let-7c, miR-486-5p, miR-183-5p, miR-185-5p, miR-106b-3p, miR-98-5p, miR-4448, miR-30c-2-5p, miR-151a-3p, miR-200c-3p, miR-21-5p, miR-10b-5p, miR-224-5p, and mir-148b-3p) as candidate NMIBC G3 biomarkers (Table 2). Plot counts are shown in Supplementary Figure 2.

In the final comparison, MIBC versus controls, 11 DEmiRNAs and 48 PPmiRNAs were found (Supplementary Table 2C). We selected ten miRNAs as candidate MIBC biomarkers (Table 2). Plot counts of these miRNAs are reported in Supplementary Figure 3.

Out of seven miRNAs selected for NMIBC G1 + G2, 15 for NMIBC G3 and 10 for MIBC, eight miRNAs were in common between the different BC subtypes with three of them in common among all three groups. Finally, 21 miRNAs were selected for the Replica/Validation phase. The complete list of all candidate miRNA biomarkers for each comparison is reported in Table 2. Heatmaps for all miRNAs emerging from all comparisons are reported in Supplementary Figure 4.

To select proper endogenous controls for qPCR normalization, data from NGS were analysed adapting the pipeline developed by Eisenberg and Levanon [14]. Two reference genes (miR-28-3p and miR-361-3p) were responding to the selection criteria and were employed as endogenous controls in the qPCR analyses.

### Replica/Validation

Twenty-one candidate miRNAs from the Discovery phase were validated by qPCR on the same set of BC cases and controls employed in the small RNA-seq analyses (Replica set) and on urine samples from additional 46 BC cases (43 G1 + G2 and 3 G3) and 16 controls (Validation set). miR-28-3p and miR-361-3p were also analyzed in the Replica/Validation as endogenous controls for normalization. miR-4448 was not detected by qPCR.

The results of Replica/Validation are reported in Table 3 and in Supplementary Table 3. The normalized expression levels from qPCR showed patterns comparable to those provided by sequencing, although with different significance. For NMIBC G1 + G2 patients, there was a significant down-regulation of miR-30c-2-5p (*p =* 0.02) and up-regulation of miR-205-5p (*p* < 0.001) in cases compared to controls (Replica set; Table 3). In the overall NMIBC G1 + G2 cases (Replica + Validation) when compared with all controls, only miR-205-5p remained significantly upregulated (*p =* 0.0001), together with miR-486-5p (*p =* 0.02) and let-7i-5p (*p =* 0.03) that were significantly up-regulated in the Discovery phase.

**Table 3:** miRNAs analysed in the replica/validation phase by qPCR and stratified for BC invasiveness and grade

		Replica			Replica/Validation		
	miRNA	Log2 Fold Change	*P*	adj P	Log2 Fold Change	*P*	adj P
MIBC	^a^miR-21-5p	0.65	0.309	0.386	0.73	0.271	0.338
	^a^miR-106b-3p	1.33	0.069	0.116	1.58	0.054	0.107
	^b^miR-30a-5p	–2.43	**0.002**	**0.011**	–2.12	**0.006**	**0.017**
	^b^let-7c-5p	–1.29	0.144	0.205	–1.04	0.234	0.312
	^b^miR-486-5p	2.55	**0.026**	0.058	2.75	**0.017**	**0.038**
	^c^miR-205-5p	1.84	**0.005**	**0.017**	1.92	**0.012**	**0.029**
	miR-451a	3.13	**0.011**	**0.031**	3.57	**0.004**	**0.014**
	miR-25-3p	1.97	**0.005**	**0.017**	2.21	**0.004**	**0.014**
	miR-7-1-5p	2.49	**0.002**	**0.011**	2.74	**0.001**	**0.012**
	miR-146a-5p	1.00	0.109	0.168	1.14	0.131	0.193
NMIBC G1 + G2	^d^miR-30c-2-5p	–1.08	**0.022**	0.144	–0.58	0.149	0.248
	^d^miR-151a-3p	–0.26	0.494	0.657	0.37	0.265	0.353
	^d^miR-30a-5p	–0.52	0.304	0.656	–0.28	0.482	0.508
	^b^let-7c-5p	–0.18	0.764	0.858	0.25	0.553	0.553
	^b^miR-486-5p	1.67	0.059	0.197	1.63	**0.017**	0.073
	^c^miR-205-5p	1.76	**0.000**	**0.007**	1.60	**0.000**	**0.002**
	let-7i-5p	0.31	0.427	0.656	0.76	**0.026**	0.076
NMIBC G3	^a^miR-21-5p	1.36	**0.005**	**0.008**	1.29	**0.007**	**0.011**
	^a^miR-106b-3p	1.67	**0.001**	**0.002**	1.94	**0.000**	**0.001**
	^b^miR-30a-5p	–0.97	0.127	0.149	–0.78	0.178	0.210
	^b^let-7c-5p	1.25	0.088	0.110	1.13	0.097	0.121
	^b^miR-486-5p	3.13	**0.001**	**0.002**	3.37	**0.000**	**0.001**
	^d^miR-30c-2-5p	–1.56	**0.001**	**0.002**	–1.19	**0.019**	**0.027**
	^d^miR-151a-3p	1.22	**0.001**	**0.002**	1.41	**0.001**	**0.002**
	miR-200c-3p	1.53	**0.000**	**0.001**	1.63	**0.000**	**0.001**
	miR-4448	na			na		
	miR-183-5p	1.96	**0.000**	**0.000**	1.98	**0.000**	**0.000**
	miR-185-5p	0.86	**0.015**	**0.021**	0.87	**0.022**	**0.029**
	miR-98-5p	0.34	0.473	0.526	0.00	0.995	0.995
	miR-148b-3p	0.09	0.887	0.887	–0.03	0.951	0.995
	miR-10b-5p	–1.69	**0.018**	**0.024**	–1.64	**0.005**	**0.008**
	miR-224-5p	2.76	**0.000**	**0.000**	2.97	**0.000**	**0.000**

Significant results in bold. Abbreviations: FDR false discovery rate, PP predictive power, MIBC muscle-invasive bladder cancer, NMIBC non-muscle invasive bladder cancer.

^a^miRNAs in common between NMIBC G3 and MIBC.

^b^miRNAs in common among NMIBC G1 + G2, NMIBC G3, and MIBC.

^c^miRNAs in common between NMIBC G1 + G2 and MIBC.

^d^miRNAs in common between NMIBC G1 + G2 and NMIBC G3.

Among NMIBC G3 patients and controls, out the 15 miRNAs tested by qPCR, ten resulted significantly differentially expressed in both Replica and Replica + Validation sets. More specifically, miR-21-5p, miR-106b-3p, miR-486-5p, miR-151a-3p, miR-200c-3p, miR-183-5p, miR-185-5p, and miR-224-5p (*p*-value in Replica + Validation set ranging from 1.94 × 10^–6^ to 0.02) resulted upregulated in NMIBC G3, while miR-30c-2-5p and miR-10b-5p were down-regulated (*p =* 0.02 and *p =* 0.005, respectively; Table 3).

For MIBC, miR-486-5p, miR-205-5p, miR-451a, miR-25-3p, and miR-7-1-5p resulted significantly upregulated in comparison to controls (*p*-value ranging from 0.001 to 0.02) while miR-30a-5p (*p =* 0.006) was down-regulated (Replica + Validation set, Table 3). Results from small RNA-seq for MIBC were further compared with data from the The Cancer Genome Atlas (TCGA) dataset [15]. Out of 324 MIBC cases of Caucasian descent with available miRNA expression quantification, only 16 had both “Solid Tissue Normal” and “Primary Tumor” samples available. Seven miRNAs found in the Discovery phase for MIBC were also significantly differentially expressed in the same direction in TCGA dataset (Table 4).

**Table 4:** DEmiRNAs found in the discovery phase for MIBC analysed in the TCGA dataset

ID miRNA (present study)	ID miRNA (TCGA)	Mean read counts	log2 Fold Change	adj P (Bonferroni)	adj P (FDR)
miR-30a-5p	miR-30a	179888	–2.496	**5.28045E–21**	**1.26467E–19**
miR-21-5p	miR-21	854585	1.717	**9.28451E–09**	**1.9336E–08**
miR-106b-3p	miR-106b	2614	1.091	**9.0971E–06**	**7.14346E–06**
let-7c-5p	let-7c	27205	–1.896	**0.0002**	**8.76786E–05**
miR-7-1-5p	miR-7-1	93	0.968	**0.0005**	**0.0002**
miR-205-5p	miR-205	25289	1.956	**0.0049**	**0.0015**
miR-25-3p	miR-25	47417	0.656	0.0801	**0.0165**
miR-486-5p^#^	miR-486-2^#^	475	–0.645	1.8177	0.2466
miR-486-5p^#^	miR-486-1^#^	473	–0.593	2.3635	0.3102
miR-451a	miR-451a	3849	–0.221	6.7117	0.7409
miR-146a-5p	miR-146a	482	–0.058	8.8212	0.9087

Abbreviations: FDR false discovery rate, MIBC muscle-invasive bladder cancer.

Significant results in bold.

^#^for this miRNA it was not possible to distinguish the locus.

**Figure 1:**
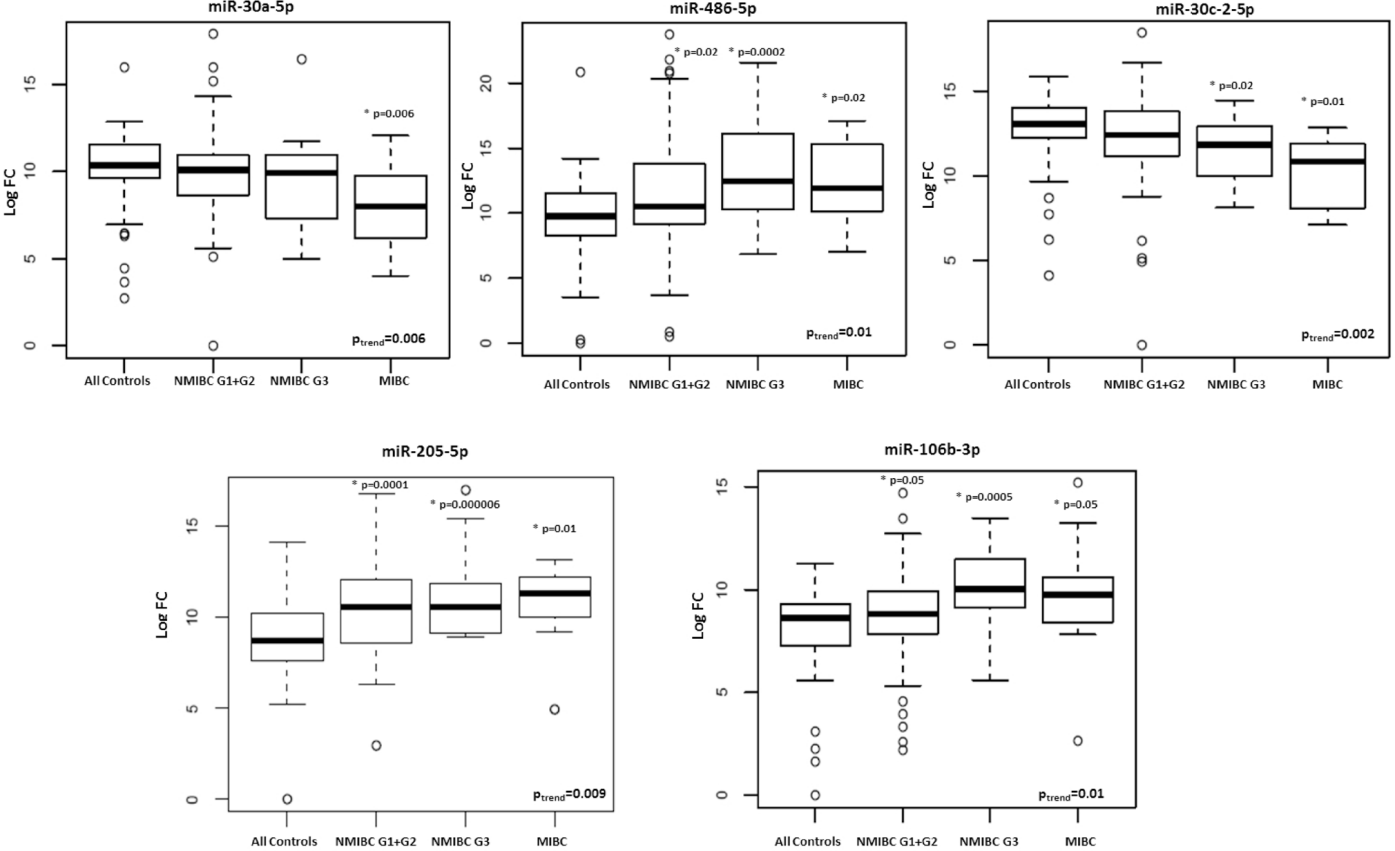
Box plots of expression levels of selected miRNAs with a significant trend (adjusted *p*-value < 0.05) from healthy controls to MIBC patients

We observed a significant trend of increasing/decreasing expression levels from healthy controls to MIBC patients for two out of three miRNAs (miR-30a-5p and miR-486-5p) that were tested in the 3 BC subtypes (*p =* 0.006 and 0.01, respectively). Significant trends were observed also for some miRNAs in common between NMIBC G1 + G2 and G3 (miR-30c-2-5p; *p =* 0.002), between NMIBC G1 + G2 and MIBC (miR-205-5p; *p =* 0.009) and NMIBC G3 and MIBC (miR-106b-3p; *p =* 0.01) (Supplementary Table 4; Figure 1). We conducted the same analyses considering only BC cases and we confirmed a significant decreasing trend for miR-10b-5p, miR-98-5p, miR-148-3p, miR-30a-5p and miR-30c-2-5p (*p*-value from 0.002 to 0.04).

**Figure 2:**
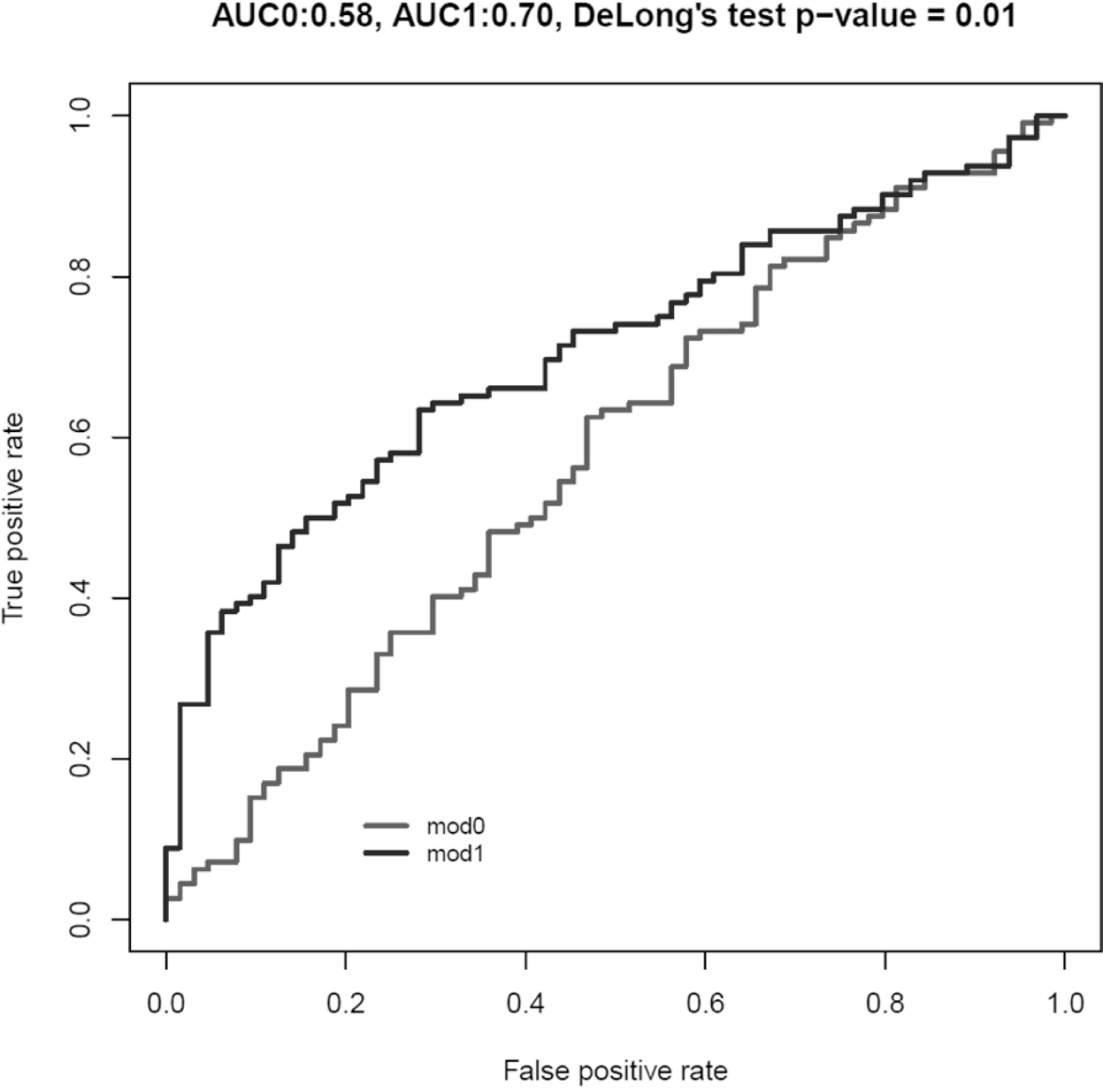
Area under the ROC curve (AUC) for Model 0 (including age and smoking habit as risk category; in grey) and Model 1 (including Model 0 plus expression levels of miR-30a-5p, let-7c-5p and miR-486-5p; in black)

Finally, we designed two models: Model 0 including traditional BC risk factors (age and smoking status) and Model 1 (i.e., Model 0 plus miR-30a-5p, let-7c-5p and miR-486-5p expression levels in the three BC subtypes). Model 1 showed a statistically significant improvement in the discrimination of cases and controls in comparison with Model 0 (Area under the ROC curve (AUC) model 0 = 0.57, 95% CI: 0.49–0.67; AUC model 1 = 0.70, 95% CI: 0.63–0.78, DeLong’s test *p =* 0.01, Figure 2). Considering the 3 BC subgroups separately and including in each Model 1 only significant DEmiRNAs from Table 3, we observed a statistically significant improvement in the discrimination of for each subgroup of cases and controls (for NMIBC G1 + G2 AUC model 0 = 0.62; AUC model 1 = 0.73, DeLong’s test *p =* 0.02; for NMIBC G3, AUC model 0 = 0.57; AUC model 1 = 0.95, DeLong’s test *p =* 1.15 × 10^–6^; for MIBC, AUC model 0 = 0.64; AUC model 1 = 0.99, DeLong’s test *p =* 2.27 × 10^–5^) (data not shown).

Each of the selected miRNAs shows a large number of validated target genes (ranging from 66 to 913) which resulted significantly involved in several important KEGG (Kyoto Encyclopedia of Genes and Genomes) pathways such as MAPK signaling (for let-7c-5p, miR-10b-5p, miR-200c-3p), ErbB signaling pathway (for let-7c-5p, miR-106b-3p), and pathways in cancer/bladder cancer. Specifically, target genes of miR-30a-5p, miR-486-5p, miR-30c-2-5p, miR-205-5p, and miR-106b-3p whose expression levels showed a trend from healthy controls to MIBC patients displayed an over-representation in several KEGG pathways such as “Pathways in cancer_Homo sapiens” (hsa05200; adj *p =* 1.05 × 10^–7^), “MicroRNAs in cancer_Homo sapiens” (hsa05206; adj *p =* 2.97 × 10^–7^) and “Bladder cancer_Homo sapiens” (hsa05219; adj *p =* 3.38 × 10^–5^).

## DISCUSSION

In the present study, we tested urinary miRNAs as non-invasive biomarkers for BC at diagnosis, with a potential application also for patients follow up. It was possible to define and validate a panel of miRNA markers that can accurately detect BC and differentiate its subtypes. Moreover, most of the miRNAs identified in the discovery set by NGS were also confirmed in the Replica + Validation set by qPCR.

Individual miRNA profiles may provide a low accuracy as cancer biomarkers, mostly due to the multifactorial nature of BC but also the large number of targets for a single miRNA [16]. Therefore, the combination of specific miRNA profiles may provide more robust results. Introducing miR-30a-5p, let-7c-5p and miR-486-5p altered in the 3 BC subtypes in a model for case-control discrimination, we observed a statistically significant improvement in AUC discrimination between BC and controls (from 50% to 70%). Currently, there are no validated non-invasive markers able to identify BC presence and subtypes. Therefore, the diagnosis and follow-up still rely on cystoscopies, with a huge burden for the health system and inconvenience for BC patients [5, 6]. Recently, some urine-based tests have been approved for the clinical practice. However, there is still large disparity of sensitivity and specificity across different BC grades with a still sub-optimal clinical utility [17, 18]. Since urine is in direct contact with the tumor, it represents an ideal source for investigation of non-invasive BC biomarkers. Our model based on specific miRNA profiles might represent a promising advancement in the non-invasive diagnosis of BC. Even if an accuracy of 0.7 remains still sub-optimal for a definitive implementation in the clinical practice, it surely constitutes a step in the right direction towards a less aggressive follow-up of BC patients.

Several DEmiRNAs have been repeatedly associated with BC in tissues but in general on small-size study populations [13, 19]. Interestingly, 6 miRNAs that we have also validated (miR-205-5p, miR-25-3p, miR-7-1-5p, miR-183-5p, miR-185-5p and miR-224-5p) were reported dysregulated by BC tumor tissues versus normal bladder mucosa by the Bladder Cancer Cluster Knowledge Base (http://www.bccluster.org/). This is of particular importance since the expression levels measured in bladder tissues and urine showed the same behaviour, highlighting the importance of these miRNAs as possible biomarkers for BC diagnosis.

The studies so far have been based on a candidate-driven approach (by qPCR) and relatively small sample sets, providing often mixed results (reviewed by [20] and [13]). Moreover, there is still ambiguity regarding the best type of specimen to use for miRNA investigation among whole urine, sediments, supernatant or exosomes. The majority of urinary miRNAs originates from renal and urethral cells and analysis of these cells can provide a measure of the health status of the excretory system [21]. A meta-analysis reported that urine supernatant-based studies are more reliable than urine sediment-based assay or voided urine [16]. In support of the urine supernatant-based approach, when miRNA expression profiles in four biological specimens (including tumor tissues and cell-free urine) were compared, among the top 25 up-regulated miRNAs in NMIBC there were 13 species that resulted in common among cell-free urine and cancer tissues [18]. Interestingly, four of those up-regulated miRNAs were also found in our study. Comparing our results to those reported from candidate miRNA approach studies (reviewed by [16]), only the up-regulation of miR-183-5p was confirmed [22].

We are aware that our study is not devoid of limitations, such as the absence of an independent set of urine samples from MIBC cases, and the size of the study population in the Replica + Validation phase. However, seven of the miRNAs belonging to the signature for MIBC were also significantly differentially expressed in the same direction in the TCGA dataset (on bladder tissues), confirming that molecular alterations in BC tissues are mirrored in urine. Moreover, we analyzed our cohort using an up-to-date deep sequencing technology that permits the more complete overview of the whole miRnome available at the present without the known limitations of arrays or candidate miRNA approach.

To the best of our knowledge, this is the first study that profiled miRNAs in urine of BC patients by NGS. We attempted to implement a model and a pipeline for miRNA data processing since, although some guidelines have been published [23–25], currently there is no standardized procedure.

Part of the observed discrepancies in the outcomes from different studies on urine so far, based on microarrays or qPCR, may be due to inconsistencies in name usage when referring to a list of expressed, differentially expressed or selected miRNAs. This might lead to mistakes in name-driven comparisons of miRNA lists (such as signatures) or meta-analyses from diverse sources (papers, databases). Moreover, probes to detect mature miRNA expression in general refer to or are designed on different releases of miRBase and do not undergo a regular re-annotation according to its updates. Finally, a substantial portion of publications relies on human miRNA names without disclosing their genomic annotation, contributing to uncertainties in the literature [26]. These limitations have precluded the clinical translation of biomarkers studies in the management of BC [27]. However, with the implementation of miRNA sequencing, the analyses procedure can be repeated over time with the most updated mapping, thus overcoming this problem. In the present study, we have paid particular attention to a proper characterization of miRNA precursors and correct annotation of miRNA species.

Another key issue for the comparison of miRNA expression levels is the selection of miRNAs found to be largely invariant in a sample set (i.e. endogenous controls). As there is no consensus on suitable control for urine testing, it is suggested that quantification should be performed with an equal amount of starting total RNA or adopting other stably expressed small RNAs as normalizers. In the present study, we suggested a possible approach to identify the possible internal candidate endogenous controls from NGS data.

The adoption of deep sequencing has provided a broad overview of the altered expressed miRNAs in urine while the unsupervised classification algorithm and the computation of the PP have identified a set of putative miRNAs as urinary biomarkers. Once confirmed in additional studies on larger populations of BC cases and controls, our panel of miRNAs detectable in urine may become an effective and reproducible molecular test.

## MATERIALS AND METHODS

### Patients

The study population consisted of men recruited in the Turin Bladder Cancer Study (TBCS) [28, 29]. All subjects provided written consent to participate to the study, according to the Helsinki declaration. The study was approved by the Interhospital Ethical Board of San Giovanni Battista/C.T.O./C.R.F./Maria Adelaide hospitals (Turin, Italy) and the Institutional Review Boards of the Human Genetics Foundation. Details on patients and controls are in Table 1 and Supplementary Material and Methods.

### RNA extraction and small RNA-sequencing (small RNA-seq)

The protocol for urine collection, storage and processing together with library preparation is described in Supplementary Material and Methods and in [30]. Briefly, total RNA was extracted from urine supernatant samples (see [31]) using the Urine microRNA Purification kit (Norgen Biotek, Canada), according to the manufacturer’s standard protocol.

Small RNA transcripts were converted into barcoded cDNA libraries with the NEBNext Multiplex Small RNA Library Prep Set for Illumina (New England BioLabs, USA) and run on Illumina HiSeq2000 platform (Illumina, USA).

### miRNA quantification by qPCR

Candidate miRNA biomarkers were validated in independent urine samples using the miRCURY LNA^™^ Universal RT microRNA PCR system (Exiqon, Denmark). Reverse transcription (RT) was performed using the Universal cDNA synthesis kit II (Exiqon, Denmark) according to the manufacturer’s instructions with the addition of one spike-in (UniSp6) to the RT reaction (Supplementary Material and Methods).

### Computational and statistical analyses

Raw reads quality-check, adapter clipping and mapping was performed as in [32]. After reads mapping, a matrix of integer values called count matrix was created. The value in the *i*-th row and the *j*-th column of the matrix reports how many reads have been unambiguously assigned to mature miRNA *i* in the sample *j*. The unwanted variation present in the data (e.g. batch effects) was estimated using the functions implemented in the SVA package [33](details in Supplementary Material and Methods).

miRNA expression levels were measured in cancer subtypes (MIBC, NMIBC G1 + G2 and NMIBC G3) versus controls. Candidate miRNAs were selected by a tailored pipeline adapted from [34]. In details, two statistical methods, both running on the original miRNA counts matrix, were applied: (1) identification of differentially expressed miRNAs by DESeq2 Bioconductor’s package [35]; (2) computation of a regression model in which single variable levels (i.e. individual miRNA expression levels) are used to predict the class label (i.e. BC patients or controls) of each subject (predictive power calculation [36]).

From the first method, the candidate miRNAs (DEmiRNAs) were those associated with adjusted False Discovery Rate (FDR) ≤0.05 and the mean read count ≥300. From the latter method, the candidate miRNAs (PPmiRNAs) were associated with a predictive power ≥0.70. Due to the still high number of resulting putative candidate miRNAs for the Replica/Validation, an inspection of the plot counts of miRNAs across the classes (cancer and healthy sample) was also performed. Finally, miRNAs resulting relevant from the literature were also considered. Details in Supplementary Material and Methods.

Endogenous control for qPCR normalization were identified adapting the pipeline developed by Eisenberg and Levanon [14]. Briefly, miRNAs from NGS data were selected considering the individual raw count and according to the following criteria: 1) at least 2 reads for each sample; 2) a log2 standard deviation value < 12; 3) a log2 fold change ranging between –4 and 7.

Delta Ct (DCt) values were obtained by normalizing the data to the identified endogenous controls selected from NGS outcomes. Differential miRNA expression levels (expressed as fold-change and calculated as log2^-DDCt^) between BC subtypes and controls were assessed by logistic regression adjusting for age and smoking. Results with *p*-value < 0.05 were deemed statistically significant (see Supplementary Material and Methods).

miRNA target genes were retrieved by miRWalk2.0 database [37]. EnrichR was used for gene ontological analysis and pathway enrichment [38, 39]. The open-access dataset of MIBC individuals from TCGA was also used for comparative analysis. All computational and statistical analyses are detailed in **S**upplementary Material and Methods.

## SUPPLEMENTARY MATERIALS FIGURES AND TABLES







## Abbreviations

BC: Bladder cancer
NMIBC: non-muscle-invasive bladder cancer
MIBC: muscle-invasive bladder cancer
miRNA: microRNA
NGS: next-generation sequencing
qPCR: real-time quantitative PCR
DEmiRNAs: differentially expressed miRNAs
PP: Predictive Power
small RNA-seq: small RNA-Sequencing
TCGA: The Cancer Genome Atlas
AUC: Area under the ROC curve
KEGG: Kyoto Encyclopedia of Genes and Genomes
TBCS: Turin Bladder Cancer Study
cDNA: complementary DNA
RT: Reverse transcription
FDR: False Discovery Rate
DCt: Delta Ct
