# Supplementary material for: microRNA profiles in urine by next-generation sequencing can stratify bladder cancer subtypes
Source: Oncotarget. 2018 Apr 17;9(29):20658–69. doi: 10.18632/oncotarget.25057 (PMC5945522; doi:10.18632/oncotarget.25057)
Supplement: Supplementary file 1 [file oncotarget-09-20658-s001.pdf]

microRNA profiles in urine by next-generation sequencing can stratify bladder cancer subtypes

SUPPLEMENTARY MATERIALS

Study design and patients

The study population included men recruited between the years 1994–2008 to participate to the Turin Bladder Cancer Study (TBCS) [1, 2] who donated an aliquot of blood and urine. Patients were all newly diagnosed, histologically confirmed cases of BC registered at two Urology Departments of A.O.U. Città della Salute e della Scienza, in Turin (Italy). Controls were men recruited daily in random fashion from patients treated at the same urology departments for non-neoplastic disease (prostatic hyperplasia, cystitis, and others) or from patients treated at the medical and surgical departments for hernias, vasculopathies, diabetes, heart failure, asthma, or other benign diseases. Patients with cancer, liver or renal diseases, and smoking-related conditions were excluded. All subjects were informed and provided written consent to participate in the study and approve the use of their biological samples for the analyses, according to the Helsinki declaration. This study was approved by the Institutional Review Boards of the Human Genetics Foundation.

Cases were interviewed to obtain information on demographics, cancer risk factors (e.g., detailed family history of cancer), and clinical information, including the type of therapy (e.g., Bacillus Calmette–Guerin (BCG), chemotherapy, and radiotherapy). Patients with BC were followed by urologists with periodic cystoscopic examinations.

Details on patients are shown in Table 1.

Samples processing and RNA extraction

Urine samples from each participant were collected in the morning, stored at 4° C until the processing consisting of a centrifugation at 3,000 g for 10 min. The urine supernatant aliquots were then transferred in tubes and centrifuged again at 12,000 g for 10 min at 4° C and stored at –80° C until use.

Total RNA was extracted with Urine microRNA Purification kit (Norgen biotek corp, Canada), starting from 1ml of urine and according to the manufacturer’s standard protocol. The extracted RNA was eluted with 30 µL of RNase-free water. RNA quantity was determined by Qubit® 2.0 Fluorometer with Qubit® microRNA Assay Kit (Life technologies, Italy).

The study was conducted in two phases. In the Discovery phase, expression of miRNAs in urine from BC cases and controls matched for age and smoking was evaluated by small RNA-sequencing (small RNA-seq). In the Replica/Validation phase, miRNAs that were differentially expressed in the Discovery phase were further validated on the same set of BC cases and controls (Replica) and on urine samples from independent group of cases and controls (Validation) using individual microRNA LNA PCR primer sets (Exiqon, Denmark).

RNA library preparation and sequencing

For sequencing, the small RNA transcripts were converted into barcoded cDNA libraries as previously described by us [3]. Library preparation was performed with the NEBNext Multiplex Small RNA Library Prep Set for Illumina (New England BioLabs Inc., USA) followed by small RNA-seq analysis on the Illumina HiSeq2000 platform (Illumina Inc., USA). For each library, 6 µL of small RNA (~100 ng of RNA) was used in all the experimental procedures as starting material. Each library was prepared with a unique indexed primer so that the libraries could all be pooled into one sequencing lane (each pool including 24 samples). Multiplex adaptor ligations, reverse transcription primer hybridization, reverse transcription reaction and the PCR amplification were processed with regard to the protocol for library preparation (Protocol E7330, New England BioLabs Inc., USA). After PCR pre-amplification, the cDNA constructs were purified with the QIAQuick PCR Purification Kit (Qiagen, Germany) following the modifications suggested by the NEBNext Multiplex Small RNA Library Prep Protocol and loaded on the Bioanalyzer 2100 (Agilent, Germany) using the DNA High Sensitivity Kit (Agilent, Germany) according to the manufacturer's protocol.

Further quality control check and gel size selection was performed using Agencourt AMPure XP Beads (Beckman Coulter, Italy) according to the NEBNext Multiplex Small RNA Library Prep Protocol.

A concluding Bioanalyzer 2100 run with the High Sensitivity DNA Kit (Agilent, Germany) completed the workflow of library preparation. The obtained sequence libraries were subjected to the Illumina sequencing pipeline, passing through clonal cluster generation on a

single-read flow cell by bridge amplification on the cBot and 50 cycles sequencing-by-synthesis on the HiSeq2000 (Illumina Inc., USA).

miRNA quantification by quantitative reverse transcriptase polymerase chain reaction (qPCR)

Candidate miRNA biomarkers selected in the Discovery phase were further validated in independent urine samples by real-time quantitative PCR (qPCR) using the miRCURY LNA™ Universal RT microRNA PCR system (Exiqon A/S, Vedbaek, Denmark). Reverse transcription (RT) was performed using the Universal cDNA synthesis kit II (Exiqon) according to the manufacturer's instructions with the addition of one spike-in (UniSp6) to the RT reaction. For qPCR, complement cDNA was diluted 1:40. Four ul of 1:40 water diluted cDNA products were mixed at 5 ul of ExiLENT SYBR Green Mastermix and 1 ul of specific miRNA probe (Exiqon). All cDNA products were prepared in duplicate PCR reactions following manufacturer's instructions. For quality control purpose, one RNA sample was measured twice and a sample containing nuclease-free water and carrier RNA was profiled as negative control. All the reactions were run on a ABI Prism 7900 Sequence Detection System (Applied Biosystems, Foster City, CA, USA). A melt curve analysis was performed for amplification specificity of each individual target per sample.

Computational analysis

miRNA sequencing analyses

The obtained FASTQ files were quality-checked using FastQC software (http://www.bioinformatics.babraham.ac.uk/projects/fastqc/). The base quality and the N content features were also considered: all files passed both these checks.

Small-RNA-seq analysis was as described in [4]. Briefly, reads shorter than 14 nucleotides were discarded from the analysis; the remaining reads were clipped from the adapter sequences using Cutadapt software (http://cutadapt.readthedocs.io/en/stable/index.html). In Cutadapt, the maximum error rate in terms of mismatches, insertions and deletions was set to 0.15. The trimmed reads were mapped against the precursor miRNA sequences downloaded from miRBase (Release 21) using the Shrimp algorithm [5] setting the options for miRNA sequences alignment. The use of precursor miRNAs as reference guarantees a precise and specific count detection. Only those reads with maximum 2 mismatches were retained. After reads filtering steps a matrix of integer values called *count matrix* was created. The value in the *i*-th row and the *j*-th column of the matrix reports how many reads have been unambiguously assigned to mature miRNA *i* in the sample *j*.

Due to the high variability among BC subtypes (i.e. invasive and non-invasive, but also non-invasive according to grade), we initially compared miRNA expression levels in healthy subjects with respect to MIBC, NMIBC G1+G2 and NMIBC G3 categories.

The candidate miRNAs were selected by uniform (ad-hoc) pipeline of analysis (adapted from [6]) composed by two statistical methods both running on the original miRNA counts matrix: (i) identification of differentially expressed miRNAs by negative binomial generalized linear models implemented in DESeq2 package in R (version 1.6.3); (ii) computation of a regression model in which single variable levels (i.e. individual miRNA expression levels) are used to predict the class label (i.e. BC patient or healthy control) of each subject.

Differentially expressed miRNAs

The identification of DEmiRNAs was performed by DESeq2 package [7] (version 1.6.3) developed in the Bioconductor suite [www.bioconductor.org] through R statistical programming language (version 3.1.1).

The hidden variation that might be affecting part of the differential expression levels of miRNAs in the dataset due to batch effect was detected by the SVA package [8] (version 3.18). The number of surrogate variables estimated from SVA package considered in the further statistical analysis was equal to the number of batches.

The hypothesis tests used in DESeq2 was based on the likelihood ratio test (LRT). The LRT is useful for testing multiple terms at once; the terms considered in the present study were the following confounding variables: smoke, age, the surrogate variables identified by SVA package, and the class of samples (i.e. healthy or BC).

Identification of miRNAs associated with high predictive power

The identification of a set of miRNA predictors is based on the computation of a regression model in which single variable levels (i.e. miRNA expression values) are used to predict the class label (i.e. cancer or healthy) of each subject [9].

Let N be the number of subjects with $n + m = N$, where n being the number of subjects belonging to class 0 (C0, i.e. cancer samples) and m the number of subjects belonging to class 1 (C1, i.e. healthy samples). Let also J be the number of miRNAs in the *count matrix.* The Xj = $\{ x_{j1}, \ldots, x_{jN}\}$, j ϵ $\{1, \ldots, J\}$ indicate the expression of the j-th miRNA across all subjects and with $\overline{y}$ the ordered N dimensional vector of true classification labels, where $\{\overline{y}_i\}_{i=1}^{n}=0$, and $\{\overline{y}_i\}_{i=n+1}^{N}=1$.

Given the expression of the *j*-th independent variable X*j*, it is possible to predict the class (i.e. C0 or C1) to which the subject belongs.

The following equation shows the model of logistic regression used:

$$logit(p_i) = ln\left(\frac{p_i}{1-p_i}\right) = \beta_0 + \beta_j X_{ji}$$

where pi is the predicted probability of success for subject i, β_0 the intercept of the model, β_j the fitted parameter and Xji the expression of the j-th gene of subject i. The logistic-regression fit leads to J N-dimensional vectors p of predicted probabilities of success, where each component is the pi calculated by the previous equation. Since the possible class labels are only cancer subject (C0) and healthy subject (C1), the classification vector $\hat{y}$, predicted using the j-th gene as independent variable, is obtained by applying the following criterion:

$$\widehat{y_i} = \begin{cases} 1 \; if \; p_i \geq 0.5 \\ 0 \; else \end{cases}$$

The comparison of $\hat{y}$ with $\bar{y}$ measures the ability of each predictor to correctly classify the subjects. This quantity is called predictive power (PP) and is defined as follows:

$$PP_j = \frac{\sum_{i=1}^{N} 1_{\{\hat{y}_i = \bar{y}_i\}}}{N}, \quad \forall j \in \{1, \ldots J\}$$

The J values of PP form a distribution of predictive power values, describing the ability of the DEGs of classifying the samples. The P (with P ≤ J) good predictors miRNAs are chosen among those miRNAs whose PP ≥ q, with q being a quantile of the predictive powers distribution, describing the minimal number of subjects a miRNAs needs to correctly classify to be considered a good predictor.

Selection of candidate miRNAs

The methodologies to determine the differential expressed miRNAs and the computation of the predictive power are applied on the miRNA counts matrix and the results are selected according the following criteria. From the first method, the candidate miRNAs (DEmiRNAs) are those associated with adjusted FDR≤0.05 and with the mean read count ≥300. From the latter method, the candidate PPmiRNAs are associated with a predictive power greater than 0.70.

The final selection of candidate miRNAs for the Replica/Validation step was based on the common miRNAs between the list of candidate miRNAs identified by both the two statistical methods. However, a careful examination of the reads counts for a single miRNA across the classes (cancer and healthy samples) was performed on all the common miRNAs and the best DEmiRNAs and PPmiRNAs in order to select those having a clear separation of the counts distribution between the two classes.

Finally, for the sake of completeness we also take into account miRNA biomarkers already reported in literature, as an additional validation of our results. In particular, miR-106b was also included in the validation step since it has been indicated as a good biomarker for BC in several papers[10–12].

Selection of miRNAs as endogenous controls from NGS data

The selection of endogenous miRNA controls for normalizing miRNA expression in the qPCR analysis for the Replica/Validation phases was performed using the approach developed by Eisenberg and Levanon [13] and adapted to miRNA NGS data.

In details, miRNAs were selected considering the individual raw count and the following criteria: (i) the single miRNA expression must be observed in all samples (at least 2 reads for each sample), (ii) with a low variance between samples (log2 standard deviation value <12), and (iii) no outstanding expression in any sample (log2 fold change between –4 and 7).

Two reference genes (miR-28-3p and miR-361-3p) were responding to the selection criteria and were employed as endogenous controls in the qPCR analyses.

External validation in TCGA MIBC tumor and normal tissues

Results from the Discovery phase between controls and MIBC cases were further compared with an open-access dataset of MIBC individuals from The Cancer Genome Atlas (TCGA) project (for a detailed description of the generation of data see [14]).

The differential expression analysis of the raw count data between MIBC tumor and normal tissues from the same subjects was performed using DESeq2 package. P-values adjusted for multiple testing (False Discovery Rate, FDR and Bonferroni`s) were considered as statistically significant.

Analysis of target genes

For selected miRNAs, the set of validated target genes was extracted by the miRWalk database [15]. EnrichR was used for gene ontological analysis and pathway enrichment [16, 17].

The list of target genes for each selected miRNA was also filtered with the list of genes dysregulated in BC as reported in the BC Cluster database (http://www.bccluster.org/). The resulting genes in common were tested for their over-representation using EnrichR.

The relevance of each gene set enrichment was estimated using a p-value adjusted for multiple testing

based on hypergeometric distribution. Gene sets with a probability values under 5% were considered significantly overrepresented.

Analysis of qPCR data

GenEx software (Multi-D) was used for data pre-processing including inter-plate calibration, evaluation of isolation and reverse transcription efficiency, setting specific cut-offs for negative control miRNA Ct values, and duplicates averaging. The analyses were performed calculating delta Ct (DCt) values either by global mean normalization or normalization according to the two reference genes (miR-28-3p and miR-361-3p) selected from NGS. The fold-change was calculated as log $2^{-\Delta\Delta CT}$ between BC (or BC subcategories) and control samples.

MiRNAs with a Ct value > 38 were deemed to be not detected. To avoid biased inference due to qPCR non-detects (Ct value = 40) a left-censoring approach was employed. Ct values of 40 were in fact substituted with the highest observed Ct value for a given miRNA [18]. Ct values were then normalized by subtracting the Ct value of the selected endogenous controls or the global mean Ct from each of the 21 miRNAs of interest. Differential miRNA expression was determined by logistic regression adjusted for age and smoking. The unadjusted p-values< 0.05 were considered as statistically significant, since these analyses were hypothesis-driven.

To test whether miRNA expression values exhibit an increasing or decreasing behavior from healthy controls to MIBC patients, a trend test adjusted for multiple testing (FDR) was performed. Similar analyses were conducted considering only BC cases categories (i.e. NMIBC G1+G2, NMIBC G3, MIBC).

Finally, we tested for the improvement in case-control discrimination when considering the information given by miRNA expression together with traditional risk factors. Predictive performance was assessed with respect to the ability of each miRNA and the traditional BC risk factors to discriminate between cases and controls using the Receiver Operating Curves (ROC) of the two models by the DeLong test (pROC package). To perform the ROC curve and to assess the area under the curve (AUC), functions prediction and performance from the ROCR package were used

RESULTS

Sample details

In total, 116 urine samples were included to be performed by small RNA-seq in the Discovery phase. Of these, one sample was discarded since the patient was affected by bladder hyperplasia, while another sample was discarded since the number of raw reads obtained resulted extremely low. Finally, 114 samples were used for the analyses (66 BC cases and 48 controls). Among cases, 10 resulted MIBC while 56 were NMIBC (39 G1 + G2 and 17 G3) (Table 1).

An average of 10.6 million reads were generated from the 114 libraries, ranging from 0.18 to 84.8 million reads per sample. Raw reads were trimmed for adaptor sequence: reads with length less than 14 nucleotides were discarded. This gave an average of 7.37 million reads per sample, ranging from 0.15 to 62.9 million reads (Supplementary Table 1). Out of an average of 7.37 million of trimmed reads an average of 0.65 (6%) millions of reads were mapped to the list of pre-miRNAs collected in miRBase (release 21). Considering all samples, an average of 975 (38%) unique miRNAs were identified and associated with at least one read (ranging from 302 (11%) to 1744 (68%)). After the mapping step, we created a *count matrix* composed by 114 samples and 1822 miRNAs having at least one read in one sample. We selected those miRNAs passing to the Discovery phase based on those having at least 20 counts considering all samples. 1787 out of 1822 miRNAs have been considered to the biomarkers identification.

Analysis of target genes

For those miRNAs showing a significant trend of increasing/decreasing expression levels from healthy controls to MIBC patients (miR-30a-5p, miR-486-5p, miR-30c-2-5p, miR-205-5p and miR106b-3p) the set of validated target genes was extracted with miRWalk database [15]. The full list of target genes (n = 1145) was tested for their over-representation using EnrichR. Several pathways in the Kyoto Encyclopedia of Genes and Genomes (KEGG) resulted significantly enriched. Among them of particular interest were the following: "Pathways in cancer_Homo sapiens_hsa05200" (Adjusted p-value= 0.0000001), "MicroRNAs in cancer_Homo sapiens_hsa05206" (Adjusted p-value = 0.0000003), and "Bladder cancer_Homo sapiens_hsa05219" (Adjusted p-value = 0.00003).

The list of target genes for each selected miRNA was also filtered with the list of genes dysregulated in BC as reported in the BC Cluster database (http://www.bccluster.org/). The resulting genes in common (n = 65) were tested for their over-representation using EnrichR. Several genes were over-represented in a list of KEGG pathways and again "Pathways in cancer_Homo sapiens_hsa05200" (Adjusted p-value ≤ 0.0000001), "MicroRNAs in cancer_Homo sapiens_hsa05206" (Adjusted p-value = 0.00001), and "Bladder cancer_Homo sapiens_hsa05219" (Adjusted p-value = 0.0001) resulted among the top 10 significant pathways (data available upon request).

REFERENCES

1. Turinetto V, Pardini B, Allione A, Fiorito G, Viberti C, Guarrera S, Russo A, Anglesio S, Ruo Redda MG, Casetta G, Cucchiarale G, Destefanis P, Oderda M, et al. H2AX phosphorylation level in peripheral blood mononuclear cells as an event-free survival predictor for bladder cancer. Molecular carcinogenesis. 2015; 55:1833–1842.
2. Sacerdote C, Guarrera S, Ricceri F, Pardini B, Polidoro S, Allione A, Critelli R, Russo A, Andrew AS, Ye Y, Wu X, Kiemeney LA, Bosio A, et al. Polymorphisms in the XRCC1 gene modify survival of bladder cancer patients treated with chemotherapy. International journal of cancer. 2013; 133:2004–2009.
3. Ferrero G, Cordero F, Tarallo S, Arigoni M, Riccardo F, Gallo G, Ronco G, Allasia M, Kulkarni N, Matullo G, Vineis P, Calogero RA, Pardini B, Naccarati A. Small non-coding RNA profiling in human biofluids and surrogate tissues from healthy individuals: description of the diverse and most represented species. Oncotarget. 2018; 9: 3097–3111. https://doi.org/10.18632/oncotarget.23203.
4. Cordero F, Beccuti M, Arigoni M, Donatelli S, Calogero RA. Optimizing a massive parallel sequencing workflow for quantitative miRNA expression analysis. PLoS One. 2012; 7:e31630.
5. Rumble SM, Lacroute P, Dalca AV, Fiume M, Sidow A, Brudno M. SHRiMP: accurate mapping of short color-space reads. PLoS computational biology. 2009; 5:e1000386.
6. Martina F, Beccuti M, Balbo G, Cordero F. Peculiar Genes Selection: A new features selection method to improve classification performances in imbalanced data sets. PLoS One. 2017; 12:e0177475.
7. Love MI, Huber W, Anders S. Moderated estimation of fold change and dispersion for RNA-seq data with DESeq2. Genome biology. 2014; 15:550.
8. Leek JT. svaseq: removing batch effects and other unwanted noise from sequencing data. Nucleic acids research. 2014; 42.
9. Liao JG, Chin KV. Logistic regression for disease classification using microarray data: model selection in a large p and small n case. Bioinformatics. 2007; 23:1945–1951.
10. Tutar Y. miRNA, cancer; computational and experimental approaches. Curr Pharm Biotechnol. 2014; 15:429.
11. Jin N, Jin X, Gu X, Na W, Zhang M, Zhao R. Screening biomarkers of bladder cancer using combined miRNA, mRNA microarray analysis. Molecular medicine reports. 2015; 12:3170–3176.
12. Zhou X, Zhang X, Yang Y, Li Z, Du L, Dong Z, Qu A, Jiang X, Li P, Wang C. Urinary cell-free microRNA-106b as a novel biomarker for detection of bladder cancer. Medical oncology. 2014; 31:197.
13. Eisenberg E, Levanon EY. Human housekeeping genes, revisited. Trends in genetics. 2013; 29:569–574.
14. Cancer Genome Atlas Research Network. Comprehensive molecular characterization of urothelial bladder carcinoma. Nature. 2014; 507:315–322.
15. Dweep H, Sticht C, Pandey P, Gretz N. miRWalk--database: prediction of possible miRNA binding sites by “walking” the genes of three genomes. Journal of biomedical informatics. 2011; 44:839–847.
16. Chen EY, Tan CM, Kou Y, Duan Q, Wang Z, Meirelles GV, Clark NR, Ma’ayan A. Enrichr: interactive and collaborative HTML5 gene list enrichment analysis tool. BMC Bioinformatics. 2013; 14:128.
17. Kuleshov MV, Jones MR, Rouillard AD, Fernandez NF, Duan Q, Wang Z, Koplev S, Jenkins SL, Jagodnik KM, Lachmann A, McDermott MG, Monteiro CD, Gundersen GW, Ma’ayan A. Enrichr: a comprehensive gene set enrichment analysis web server 2016 update. Nucleic acids research. 2016; 44:W90–97.
18. McCall MN, McMurray HR, Land H, Almudevar A. On non-detects in qPCR data. Bioinformatics. 2014; 30:2310–2316.

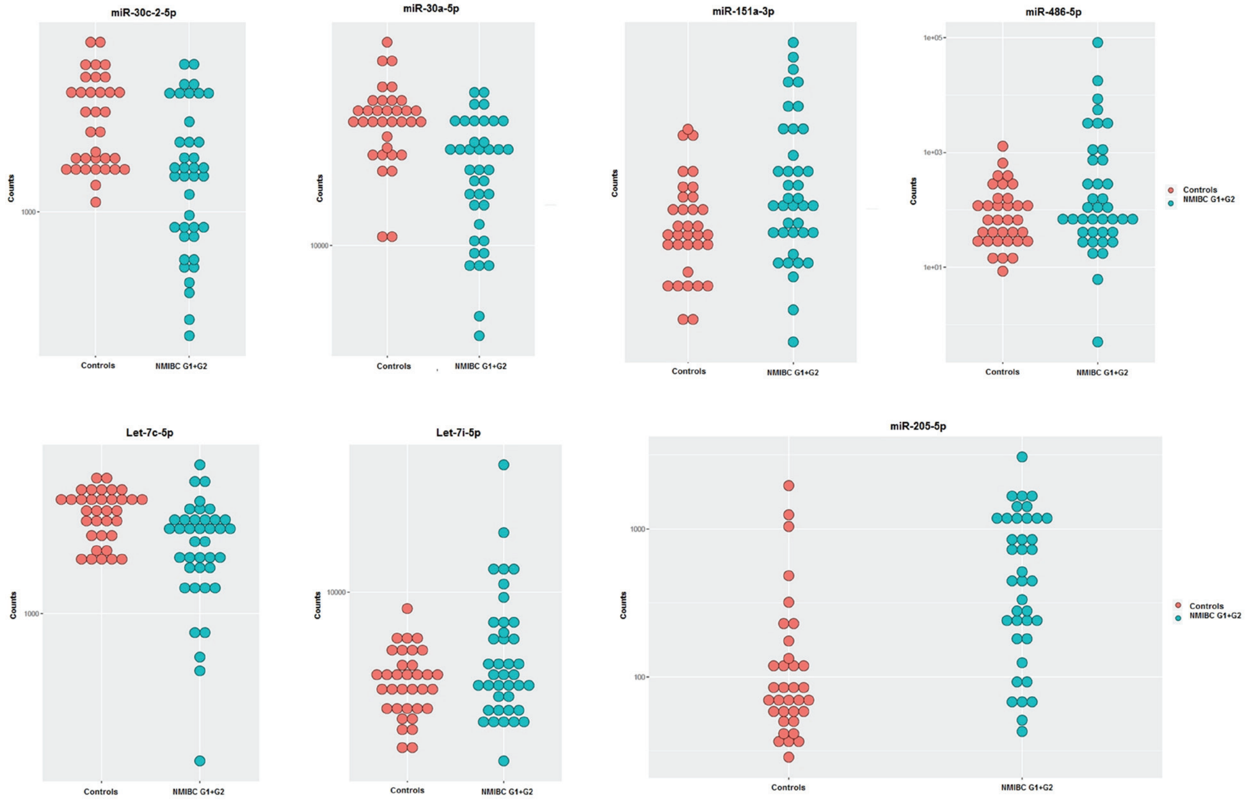

Supplementary Figure 1: Plot of read counts of selected miRNAs differentially expressed between NMIBC G1 + G2 and controls.

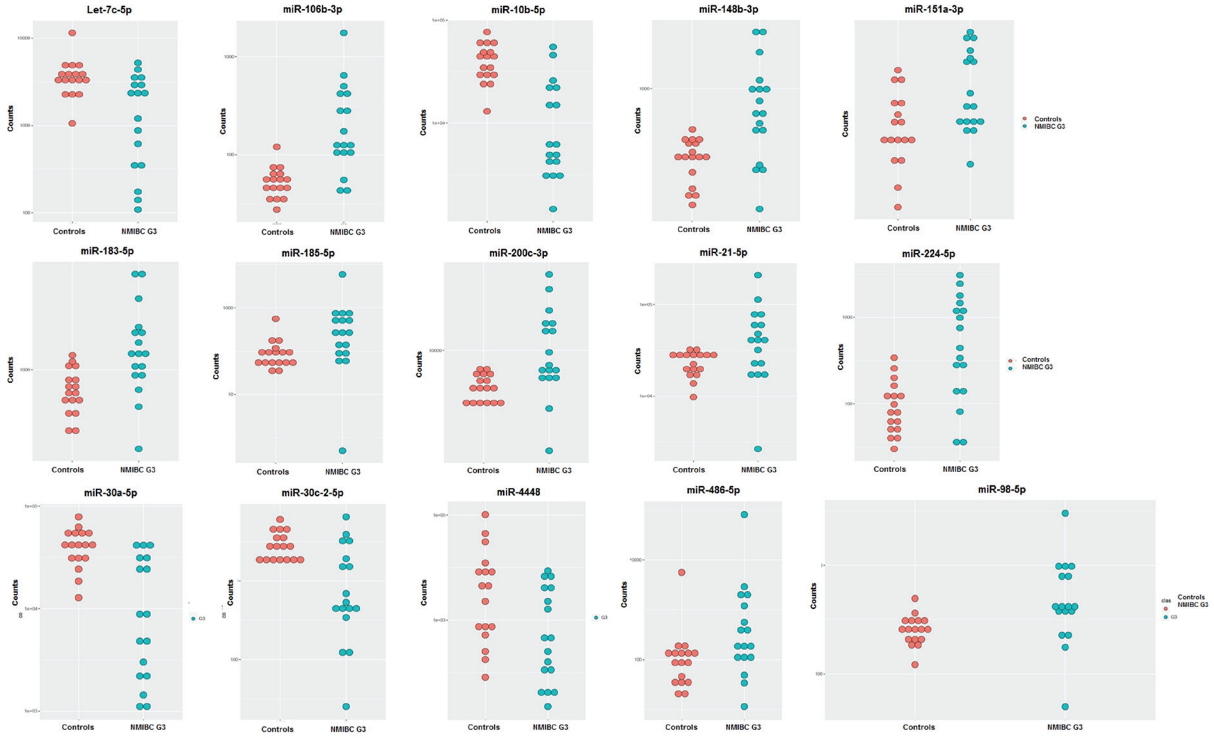

Supplementary Figure 2: Plot of read counts of selected miRNAs differentially expressed between NMIBC G3 and controls.

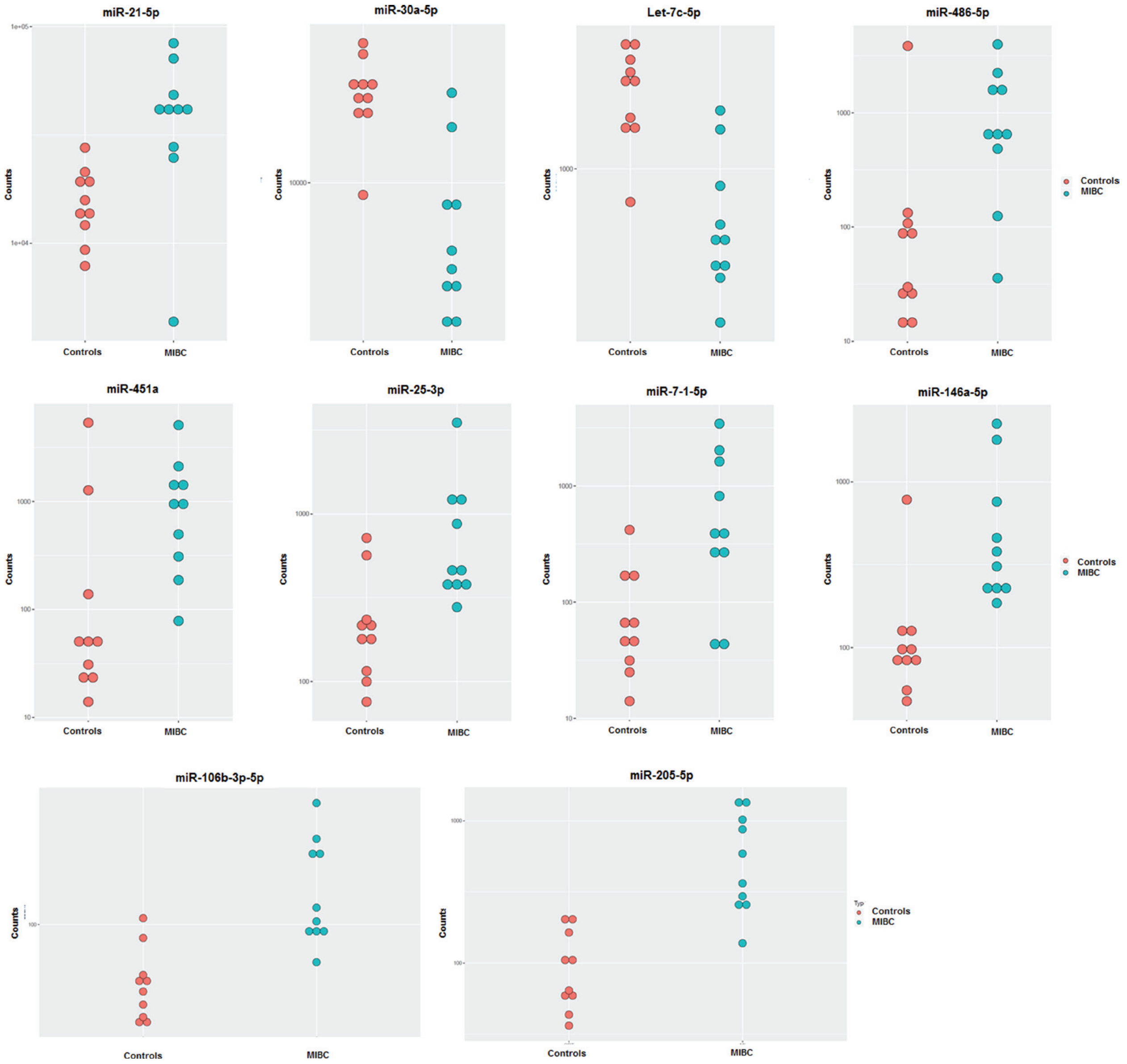

Supplementary Figure 3: Plot of read counts of selected miRNAs differentially expressed between MIBC and controls.

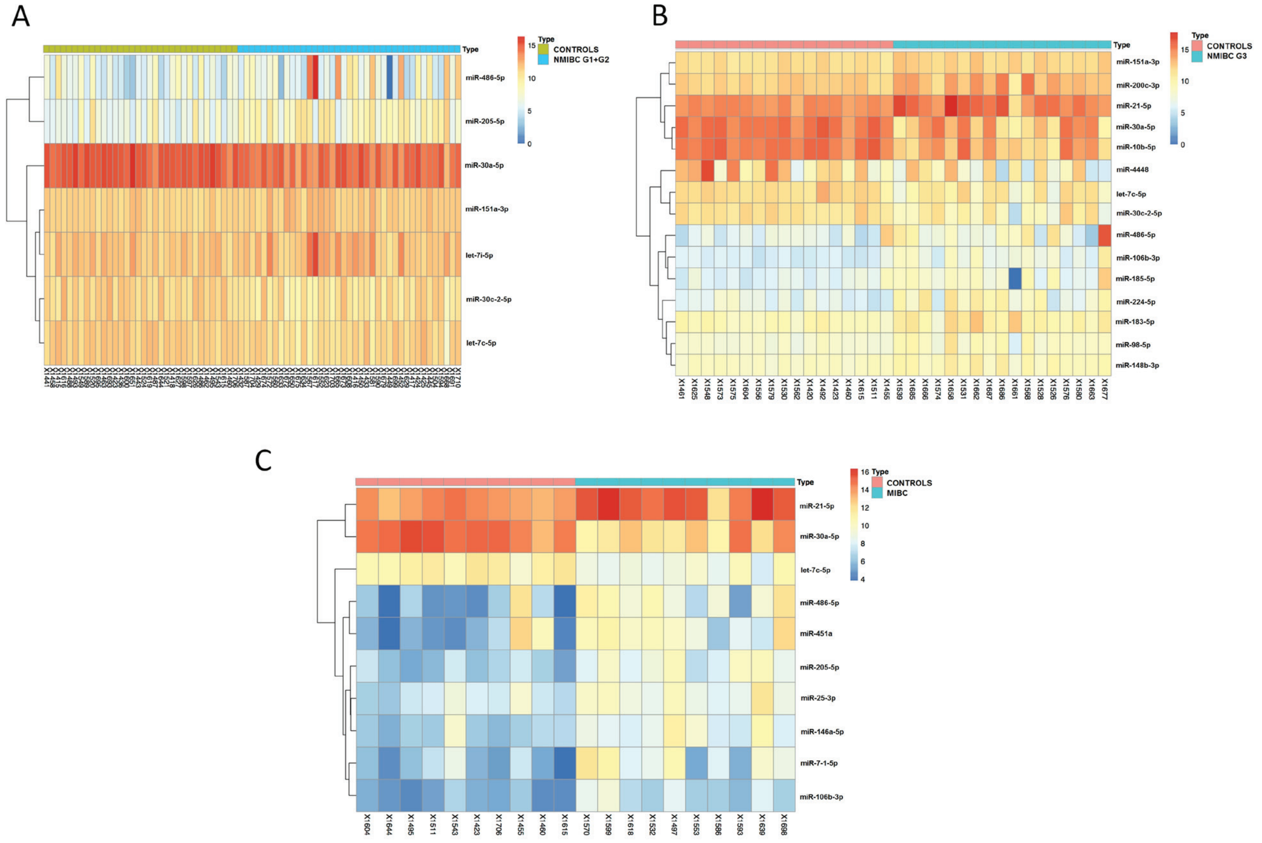

Supplementary Figure 4: Heatmap of the selected miRNAs in the Discovery phase differentially expressed between (**A**) NMIBC G1 + G2 and controls; (**B**) NMIBC G3 and controls, and (**C**) MIBC and controls.

Supplementary Table 1: Overview of raw sequences reads for each patient and sequence reads after adaptor trimming, read count, and sequence length selection. See Supplementary_Table_1

Supplementary Table 2: Urinary miRNAs from the Discovery phase resulting differentially expressed between A) NMIBC G1+G2 and controls. (B) NMIBC G3 and controls. and C) MIBC and controls. See Supplementary_Table_2

Supplementary Table 3: miRNAs analysed with qPCR in the Replica/Validation phase stratified for BC invasiveness and grade

		Replica			Validation			Overall		
	**miRNA**	**Log2 Fold Change**	***p***	**adj *p***	**Log2 Fold Change**	***p***	**adj *p***	**Log2 Fold Change**	***p***	**adj *p***
MIBC	[a]miR-21-5p	0.65	0.309	0.386				0.73	0.271	0.338
	[a]miR-106b-3p	1.33	0.069	0.116				1.58	0.054	0.107
	[b]miR-30a-5p	–2.43	**0.002**	**0.011**				–2.12	**0.006**	**0.017**
	[b]let-7c-5p	–1.29	0.144	0.205				–1.04	0.234	0.312
	[b]miR-486-5p	2.55	**0.026**	0.058				2.75	**0.017**	**0.038**
	[c]miR-205-5p	1.84	**0.005**	**0.017**				1.92	**0.012**	**0.029**
	miR-451a	3.13	**0.011**	**0.031**				3.57	**0.004**	**0.014**
	miR-25-3p	1.97	**0.005**	**0.017**				2.21	**0.004**	**0.014**
	miR-7-1-5p	2.49	**0.002**	**0.011**				2.74	**0.001**	**0.012**
	miR-146a-5p	1.00	0.109	0.168				1.14	0.131	0.193
NMIBC G1+G2	[d]miR-30c-2-5p	–1.08	**0.022**	0.144	1.30	0.142	0.190	–0.58	0.149	0.248
	[d]miR-151a-3p	–0.26	0.494	0.657	1.85	**0.011**	**0.066**	0.37	0.265	0.353
	[b]miR-30a-5p	–0.52	0.304	0.656	0.79	0.311	0.364	–0.28	0.482	0.508
	[b]let-7c-5p	–0.18	0.764	0.858	1.39	**0.039**	0.110	0.25	0.553	0.553
	[b]miR-486-5p	1.67	0.059	0.197	2.11	0.115	0.175	1.63	**0.017**	0.073
	[c]miR-205-5p	1.76	**0.000**	**0.007**	2.47	**0.007**	**0.066**	1.60	**0.000**	**0.002**
	let-7i-5p	0.31	0.427	0.656	1.72	**0.023**	0.093	0.76	**0.026**	0.076
NMIBC G3	[a]miR-21-5p	1.36	**0.005**	**0.008**	1.42	0.367	0.459	1.29	**0.007**	**0.011**
	[a]miR-106b-3p	1.67	**0.001**	**0.002**	3.09	0.102	0.353	1.94	**0.000**	**0.001**
	[b]miR-30a-5p	–0.97	0.127	0.149	–0.02	0.990	0.990	–0.78	0.178	0.210
	[b]let-7c-5p	1.25	0.088	0.110	0.47	0.803	0.875	1.13	0.097	0.121
	[b]miR-486-5p	3.13	**0.001**	**0.002**	4.51	0.135	0.353	3.37	**0.000**	**0.001**
	[d]miR-30c-2-5p	–1.56	**0.001**	**0.002**	0.40	0.831	0.875	–1.19	**0.019**	**0.027**
	[d]miR-151a-3p	1.22	**0.001**	**0.002**	1.99	0.287	0.417	1.41	**0.001**	**0.002**
	miR-200c-3p	1.53	**0.000**	**0.001**	2.02	0.292	0.417	1.63	**0.000**	**0.001**
	miR-4448	na			na	na	na	na	na	na
	miR-183-5p	1.96	**0.000**	**0.000**	2.09	0.242	0.417	1.98	**0.000**	**0.000**
	miR-185-5p	0.86	**0.015**	**0.021**	1.36	0.403	0.474	0.87	**0.022**	**0.029**
	miR-98-5p	0.34	0.473	0.526	–2.37	0.129	0.353	0.00	0.995	0.995
	miR-148b-3p	0.09	0.887	0.887	–0.86	0.273	0.417	–0.03	0.951	0.995
	miR-10b-5p	–1.69	**0.018**	**0.024**	–1.21	**0.029**	0.289	–1.64	**0.005**	**0.008**
	miR-224-5p	2.76	**0.000**	**0.000**	5.49	**0.001**	**0.021**	2.97	**0.000**	**0.000**

Significant results in bold. Abbreviations: FDR false discovery rate, PP predictive power, MIBC muscle-invasive bladder cancer, NMIBC non-muscle invasive bladder cancer.
[a]miRNAs in common between NMIBC G3 and MIBC.
[b]miRNAs in common among NMIBC G1 + G2, NMIBC G3, and MIBC.
[c]miRNAs in common between NMIBC G1 + G2 and MIBC.
[d]miRNAs in common between NMIBC G1 + G2 and NMIBC G3.

Supplementary Table 4: Trend test (adjusted *p*-value < 0.05) of increasing/decreasing miRNA expression levels from healthy controls to MIBC patients

TargetID	Beta	*p*-value	Adj *p*-value (FDR)
**miR-10b-5p**	–1.92	9.50E-05	**0.002**
**miR-224-5p**	1.95	7.54E-04	**0.008**
**miR-30c-2-5p**	–1.72	2.03E-03	**0.010**
**miR-25-3p**	1.87	2.12E-03	**0.010**
**miR-7-1-5p**	2.06	2.48E-03	**0.010**
**miR-451a**	2.83	2.98E-03	**0.010**
**miR-30a-5p**	–1.56	6.21E-03	**0.018**
**miR-205-5p**	1.47	8.85E-03	**0.022**
**miR-486-5p**	2.39	1.06E-02	**0.024**
**miR-106b-3p**	1.32	1.29E-02	**0.026**
**miR-98-5p**	–0.98	1.97E-02	**0.036**
**let-7i-5p**	0.98	3.04E-02	**0.051**
**miR-148b-3p**	–0.63	1.33E-01	0.205
**miR-146a-5p**	0.89	1.92E-01	0.264
**miR-185-5p**	0.54	2.02E-01	0.264
**miR-21-5p**	0.65	2.11E-01	0.264
**miR-183-5p**	0.56	2.37E-01	0.279
**let-7c-5p**	–0.59	3.18E-01	0.354
**miR-151a-3p**	0.14	7.59E-01	0.799
**miR-200c-3p**	0.08	8.51E-01	0.851

Significant results in bold. Abbreviations: FDR false discovery rate, PP predictive power, MIBC muscle-invasive bladder cancer.
