# Supplementary material for: microRNA profiles in urine by next-generation sequencing can stratify bladder cancer subtypes
Source: Oncotarget. 2018 Apr 17;9(29):20658–69. doi: 10.18632/oncotarget.25057 (PMC5945522; doi:10.18632/oncotarget.25057)
Supplement: Supplementary file 2 [file oncotarget-09-20658-s002.docx]

**Supplementary Table S1:** Overview of raw sequences reads for each patient and sequence reads after adaptor trimming, read count, and sequence length selection.

| ***ID*** | ***Starting Reads*** | ***After Cutadapt*** | ***Mapped*** | ***miRNA aligned reads (%)*** | ***miRNA reads*** | ***Unique miRNA*** |
| --- | --- | --- | --- | --- | --- | --- |
| *u_l2_31415* | *5358726* | *2961249* | *149465* | *5.05%* | *149465* | *724* |
| *u_l2_31416* | *12558336* | *9159296* | *1644699* | *17.96%* | *1644699* | *1238* |
| *u_l2_31418* | *31431855* | *23050341* | *1680819* | *7.29%* | *1680819* | *1439* |
| *u_l2_31429* | *30446048* | *25460826* | *946611* | *3.72%* | *946611* | *1120* |
| *u_l2_31432* | *45308981* | *23769573* | *977395* | *4.11%* | *977395* | *1209* |
| *u_l2_31433* | *15476247* | *7971738* | *1328926* | *16.67%* | *1328926* | *1086* |
| *u_l2_31436* | *23205635* | *16318373* | *498929* | *3.06%* | *498929* | *1422* |
| *u_l2_31441* | *6400444* | *3128080* | *317666* | *10.16%* | *317666* | *803* |
| *u_l2_31443* | *12551001* | *8087670* | *430501* | *5.32%* | *430501* | *808* |
| *u_l2_31450* | *8252538* | *4589557* | *647213* | *14.10%* | *647213* | *937* |
| *u_l2_31458* | *32806571* | *27451938* | *498683* | *1.82%* | *498683* | *1011* |
| *u_l2_31461* | *20713763* | *6558030* | *678349* | *10.34%* | *678349* | *1180* |
| *u_l2_31462* | *38512377* | *29540893* | *952441* | *3.22%* | *952441* | *1123* |
| *u_l2_31463* | *7640225* | *2845866* | *789092* | *27.73%* | *789092* | *1142* |
| *u_l2_31487* | *8975931* | *5496202* | *1187062* | *21.60%* | *1187062* | *1296* |
| *u_l2_31488* | *24779187* | *16219139* | *123373* | *0.76%* | *123373* | *789* |
| *u_l2_31492* | *5847993* | *3031496* | *485967* | *16.03%* | *485967* | *785* |
| *u_l2_31552* | *7920016* | *2901234* | *1205644* | *41.56%* | *1205644* | *1052* |
| *u_l2_31570* | *8948860* | *6238191* | *842973* | *13.51%* | *913280* | *1121* |
| *u_l2_31572* | *13514610* | *10914409* | *1217596* | *11.16%* | *1217596* | *1536* |
| *u_l2_31573* | *7182225* | *4493736* | *913280* | *20.32%* | *913280* | *1043* |
| *u_l2_31599* | *22199654* | *19242799* | *2953057* | *15.35%* | *2953057* | *1415* |
| *u_l2_31618* | *12422321* | *7413594* | *345718* | *4.66%* | *345718* | *1067* |
| *u_l3_51493* | *6162514* | *1631745* | *351580* | *21.55%* | *351580* | *877* |
| *u_l3_51556* | *19359430* | *12036067* | *408890* | *3.40%* | *408890* | *1428* |
| *u_l3_51575* | *11932871* | *7841216* | *432609* | *5.52%* | *432609* | *1126* |
| *u_l3_51587* | *8214359* | *3233927* | *449043* | *13.89%* | *449043* | *1024* |
| *u_l3_51590* | *5249868* | *2958169* | *255634* | *8.64%* | *255634* | *795* |
| *u_l3_51600* | *19749597* | *10576287* | *1483491* | *14.03%* | *1483491* | *1266* |
| *u_l3_51604* | *3098327* | *1143813* | *66577* | *5.82%* | *66577* | *819* |
| *u_l3_51608* | *10676599* | *6665732* | *735442* | *11.03%* | *735442* | *1280* |
| *u_l3_51616* | *4691548* | *2713565* | *508359* | *18.73%* | *508359* | *877* |
| *u_l3_51625* | *24966467* | *19034224* | *1019552* | *5.36%* | *1019552* | *1274* |
| *u_l3_51629* | *5816234* | *1721400* | *188463* | *10.95%* | *188463* | *923* |
| *u_l3_51633* | *10721334* | *7489851* | *376660* | *5.03%* | *376660* | *832* |
| *u_l3_51634* | *10509060* | *4355727* | *563641* | *12.94%* | *563641* | *1360* |
| *u_l3_51644* | *5595759* | *4685860* | *413642* | *8.83%* | *413642* | *793* |
| *u_l3_51651* | *6047017* | *4853001* | *1770783* | *36.49%* | *1770783* | *1160* |
| *u_l3_51662* | *12628709* | *10786441* | *3967900* | *36.79%* | *3967900* | *1499* |
| *u_l3_51666* | *14718410* | *9970836* | *894489* | *8.97%* | *894489* | *1335* |
| *u_l3_51673* | *3692032* | *2481230* | *739064* | *29.79%* | *739064* | *1081* |
| *u_l3_51674* | *9745877* | *8123048* | *370653* | *4.56%* | *370653* | *820* |
| *u_l3_51675* | *61909253* | *52756594* | *5211769* | *9.88%* | *5211769* | *1633* |
| *u_l3_51679* | *84815741* | *62919061* | *455144* | *0.72%* | *455144* | *1705* |
| *u_l3_51685* | *34125602* | *29150539* | *3205622* | *11.00%* | *3205622* | *1404* |
| *u_l3_51687* | *8544808* | *4869364* | *664871* | *13.65%* | *664871* | *1166* |
| *u_l3_51693* | *4780334* | *2491209* | *882733* | *35.43%* | *882733* | *820* |
| *51452* | *4295689* | *3888940* | *323090* | *8.31%* | *323040* | *798* |
| *51486* | *1361246* | *410079* | *38887* | *9.48%* | *38833* | *654* |
| *51495* | *1207021* | *336050* | *60917* | *18.13%* | *60828* | *632* |
| *51511* | *1665931* | *600034* | *112718* | *18.79%* | *112685* | *752* |
| *51520* | *1600250* | *543492* | *161256* | *29.67%* | *161175* | *734* |
| *51521* | *4355835* | *1994604* | *68109* | *3.41%* | *67947* | *620* |
| *51524* | *2998588* | *1215464* | *102976* | *8.47%* | *102875* | *756* |
| *51528* | *4898718* | *3846047* | *138759* | *3.61%* | *138689* | *856* |
| *51530* | *10074874* | *9381609* | *213347* | *2.27%* | *213243* | *774* |
| *51531* | *902399* | *501285* | *129323* | *25.80%* | *129090* | *680* |
| *51532* | *7480969* | *6565334* | *72843* | *1.11%* | *72743* | *1287* |
| *51539* | *8896937* | *8131220* | *1763546* | *21.69%* | *1763340* | *1358* |
| *51543* | *11258980* | *7594878* | *161287* | *2.12%* | *161156* | *1373* |
| *51548* | *24288216* | *21191264* | *165813* | *0.78%* | *165562* | *714* |
| *51549* | *6667090* | *2094190* | *37794* | *1.80%* | *37705* | *639* |
| *51560* | *3850079* | *907892* | *58316* | *6.42%* | *58247* | *639* |
| *51579* | *7335222* | *6315912* | *66364* | *1.05%* | *66254* | *526* |
| *51581* | *8398198* | *7011001* | *263514* | *3.76%* | *263420* | *779* |
| *51589* | *1952918* | *638590* | *153298* | *24.01%* | *153276* | *680* |
| *51598* | *8780629* | *7374260* | *134923* | *1.83%* | *134833* | *740* |
| *51623* | *7800385* | *6501226* | *344488* | *5.30%* | *344413* | *807* |
| *51627* | *2281367* | *1173817* | *28802* | *2.45%* | *28756* | *526* |
| *51658* | *10783359* | *9264753* | *507072* | *5.47%* | *506929* | *902* |
| *51665* | *1777459* | *1050039* | *44233* | *4.21%* | *44211* | *512* |
| *61417* | *2866927* | *1450731* | *149088* | *10.28%* | *149071* | *706* |
| *61423* | *3604066* | *959368* | *116296* | *12.12%* | *116274* | *665* |
| *61424* | *4336266* | *3181835* | *308698* | *9.70%* | *308681* | *792* |
| *61425* | *5580899* | *2313210* | *590056* | *25.51%* | *590042* | *1077* |
| *61445* | *3328812* | *2524581* | *510121* | *20.21%* | *510112* | *980* |
| *61504* | *2046644* | *818512* | *235704* | *28.80%* | *235700* | *696* |
| *61526* | *19056280* | *14784678* | *2092774* | *14.16%* | *2092702* | *1595* |
| *61567* | *2969985* | *1906280* | *851180* | *44.65%* | *851169* | *1162* |
| *61574* | *5844631* | *2360437* | *266446* | *11.29%* | *266413* | *968* |
| *61576* | *3291364* | *2130997* | *250373* | *11.75%* | *250359* | *796* |
| *61580* | *8337736* | *5644516* | *357438* | *6.33%* | *357404* | *902* |
| *61594* | *9260974* | *2296896* | *637784* | *27.77%* | *637741* | *1038* |
| *61617* | *3736469* | *2071870* | *814764* | *39.33%* | *814738* | *792* |
| *61619* | *10370367* | *5755521* | *214360* | *3.72%* | *214310* | *783* |
| *61663* | *4549218* | *1441959* | *149445* | *10.36%* | *149424* | *712* |
| *61668* | *15919500* | *10434191* | *797646* | *7.64%* | *797586* | *1149* |
| *61677* | *4026323* | *2437779* | *560313* | *22.98%* | *560293* | *857* |
| *61691* | *3325944* | *2107433* | *63263* | *3.00%* | *63244* | *568* |
| *61699* | *4264090* | *749619* | *90879* | *12.12%* | *90849* | *569* |
| *61703* | *12984684* | *9053544* | *418933* | *4.63%* | *418883* | *858* |
| *61704* | *4372645* | *974703* | *98692* | *10.13%* | *98659* | *625* |
| *61706* | *3112126* | *1312975* | *220685* | *16.81%* | *220663* | *684* |
| *61710* | *6230680* | *3475274* | *345132* | *9.93%* | *345078* | *844* |
| *71420* | *1384406* | *504383* | *155,650* | *30.85%* | *155650* | *668* |
| *71449* | *715721* | *564175* | *37,140* | *6.58%* | *37140* | *474* |
| *71455* | *48877092* | *34997078* | *668,018* | *1.90%* | *668018* | *1744* |
| *71460* | *3220838* | *1886799* | *92,124* | *4.88%* | *92124* | *1120* |
| *71497* | *18758392* | *18479867* | *1,100,260* | *5.95%* | *1100260* | *1462* |
| *71553* | *3103004* | *2385750* | *417,062* | *17.48%* | *417062* | *1487* |
| *71562* | *2103760* | *1183247* | *137,276* | *11.60%* | *137276* | *954* |
| *71568* | *2685500* | *2067503* | *571,382* | *27.63%* | *571382* | *1333* |
| *71586* | *5948433* | *3640328* | *20,111* | *0.55%* | *20111* | *593* |
| *71593* | *1484603* | *769600* | *338,529* | *43.98%* | *338529* | *997* |
| *71597* | *5824353* | *4265252* | *287,428* | *6.73%* | *287428* | *992* |
| *71615* | *9105745* | *8517755* | *231,727* | *2.72%* | *231727* | *703* |
| *71626* | *3424164* | *1839894* | *180,224* | *9.79%* | *180224* | *1169* |
| *71639* | *7807668* | *7291014* | *2,237,847* | *30.69%* | *2237847* | *1705* |
| *71650* | *600973* | *427719* | *48,278* | *11.28%* | *48278* | *509* |
| *71661* | *189522* | *156187* | *9,323* | *5.96%* | *9323* | *302* |
| *71672* | *308277* | *245304* | *18,108* | *7.38%* | *18108* | *330* |
| *71686* | *13012648* | *12356848* | *6,109,586* | *49.44%* | *6109586* | *1611* |
| *71695* | *2422413* | *2145448* | *141,850* | *6.61%* | *141850* | *910* |
| *71698* | *6867467* | *6384067* | *377,458* | *5.91%* | *377458* | *990* |
| ***Total*** | *1216379262* | *840160243* | *74703196* | *14.69%* | *74770403* | *111252* |
| ***Average*** | *10669994* | *7369827* | *655291* | *0.12* | *655881* | *976* |
