# Supplementary material for: microRNA profiles in urine by next-generation sequencing can stratify bladder cancer subtypes
Source: Oncotarget. 2018 Apr 17;9(29):20658–69. doi: 10.18632/oncotarget.25057 (PMC5945522; doi:10.18632/oncotarget.25057)
Supplement: Supplementary file 3 [file oncotarget-09-20658-s003.docx]

**Supplementary Table S2**. Urinary miRNAs from the Discovery phase resulting differentially expressed between A) NMIBC G1+G2 and controls. B) NMIBC G3 and controls. and C) MIBC and controls

**A**

| **DESEQ2** | | | | Prediction Power(logistic) | |
| --- | --- | --- | --- | --- | --- |
| **miRNAs** | Mean read counts | log2 Fold Change | adj p-value (FDR) | **miRNAs** | Prediction power |
| hsa-mir-30a | 27229.05 | -0.63 | 0.0043 | hsa-mir-30a | 0.73 |
| hsa-mir-200c | 5376.02 | 0.61 | 0.0262 | hsa-let-7c | 0.71 |
| hsa-mir-4448 | 5279.83 | -1.76 | 0.0177 | hsa-mir-205 | 0.73 |
| hsa-let-7i | 5038.27 | 0.55 | 0.0264 | hsa-mir-584 | 0.71 |
| hsa-mir-151a | 2873.15 | 0.34 | 0.0177 | hsa-mir-7706 | 0.71 |
| hsa-let-7c | 2644.33 | -0.43 | 0.0393 |  |  |
| hsa-mir-30c-2 | 2105.60 | -0.73 | 0.0019 |  |  |
| hsa-mir-22 | 1954.00 | 0.42 | 0.0376 |  |  |
| hsa-mir-486 | 1868.04 | 1.94 | 0.0249 |  |  |
| hsa-mir-24-1 | 956.32 | 0.64 | 0.0152 |  |  |
| hsa-mir-183 | 834.51 | 0.80 | 0.0012 |  |  |
| hsa-mir-205 | 479.07 | 1.82 | 0.0002 |  |  |
| hsa-let-7f-2 | 431.52 | -0.69 | 0.0019 |  |  |
| hsa-mir-25 | 367.45 | 0.87 | 0.0177 |  |  |
| hsa-mir-98 | 291.10 | 0.68 | 0.0177 |  |  |
| hsa-mir-103a-2 | 234.12 | 0.80 | 0.0129 |  |  |
| hsa-mir-146a | 218.29 | 0.81 | 0.0234 |  |  |
| hsa-mir-9-1 | 199.96 | -0.89 | 0.0019 |  |  |
| hsa-mir-185 | 192.87 | 1.22 | 0.0034 |  |  |
| hsa-mir-29a | 184.43 | -0.39 | 0.0454 |  |  |
| hsa-mir-4459 | 169.71 | -0.65 | 0.0234 |  |  |
| hsa-mir-23a | 121.64 | 0.96 | 0.0234 |  |  |
| hsa-mir-629 | 105.69 | 0.75 | 0.0464 |  |  |
| hsa-mir-106b | 99.24 | 1.40 | 0.0000 |  |  |
| hsa-mir-3124 | 95.78 | -0.95 | 0.0492 |  |  |
| hsa-mir-16-1 | 95.08 | 1.11 | 0.0141 |  |  |
| hsa-mir-93 | 75.17 | 0.94 | 0.0019 |  |  |
| hsa-mir-30b | 72.07 | -0.96 | 0.0038 |  |  |
| hsa-mir-125b-2 | 65.91 | -0.53 | 0.0277 |  |  |
| hsa-mir-16-2 | 62.11 | 1.07 | 0.0211 |  |  |
| hsa-mir-660 | 61.08 | -0.55 | 0.0383 |  |  |
| hsa-mir-4492 | 55.63 | 0.73 | 0.0234 |  |  |
| hsa-mir-584 | 55.30 | 1.17 | 0.0003 |  |  |
| hsa-mir-210 | 53.28 | 0.73 | 0.0186 |  |  |
| hsa-mir-425 | 46.76 | 0.84 | 0.0069 |  |  |
| hsa-mir-500a | 45.29 | -0.43 | 0.0177 |  |  |
| hsa-mir-483 | 41.82 | 3.87 | 0.0000 |  |  |
| hsa-mir-934 | 36.64 | 0.71 | 0.0033 |  |  |
| hsa-mir-6883 | 29.47 | 1.44 | 0.0033 |  |  |
| hsa-mir-206 | 27.61 | -0.89 | 0.0202 |  |  |
| hsa-mir-4706 | 27.26 | 1.34 | 0.0244 |  |  |
| hsa-mir-3180-4 | 26.95 | -1.10 | 0.0177 |  |  |
| hsa-mir-452 | 23.41 | 0.96 | 0.0454 |  |  |
| hsa-mir-4657 | 23.33 | -1.01 | 0.0328 |  |  |
| hsa-mir-7-3 | 20.90 | -1.06 | 0.0234 |  |  |
| hsa-mir-1-2 | 20.76 | -1.39 | 0.0328 |  |  |
| hsa-mir-330 | 20.16 | 0.61 | 0.0149 |  |  |
| hsa-mir-652 | 18.57 | 0.90 | 0.0186 |  |  |
| hsa-mir-765 | 17.89 | -1.15 | 0.0161 |  |  |
| hsa-mir-502 | 17.19 | -0.62 | 0.0207 |  |  |
| hsa-mir-31 | 16.80 | 1.00 | 0.0297 |  |  |
| hsa-mir-616 | 14.86 | -1.03 | 0.0129 |  |  |
| hsa-mir-1291 | 14.12 | 1.30 | 0.0311 |  |  |
| hsa-mir-558 | 13.71 | -1.18 | 0.0088 |  |  |
| hsa-mir-647 | 13.53 | -1.07 | 0.0234 |  |  |
| hsa-mir-3656 | 12.01 | 0.77 | 0.0161 |  |  |
| hsa-mir-1253 | 11.71 | -1.11 | 0.0398 |  |  |
| hsa-mir-503 | 11.68 | 1.20 | 0.0066 |  |  |
| hsa-mir-7706 | 11.55 | 1.21 | 0.0003 |  |  |
| hsa-mir-421 | 10.36 | 0.71 | 0.0262 |  |  |
| hsa-mir-551a | 8.82 | -1.53 | 0.0016 |  |  |
| hsa-mir-577 | 8.67 | -1.15 | 0.0311 |  |  |
| hsa-mir-4740 | 8.35 | -1.20 | 0.0393 |  |  |
| hsa-mir-5100 | 7.75 | -2.03 | 0.0002 |  |  |
| hsa-mir-151b | 7.71 | -1.02 | 0.0073 |  |  |
| hsa-mir-4302 | 7.57 | -1.14 | 0.0089 |  |  |
| hsa-mir-1236 | 6.86 | -3.24 | 0.0016 |  |  |
| hsa-mir-3972 | 6.22 | -1.92 | 0.0002 |  |  |
| hsa-mir-3916 | 6.18 | -0.80 | 0.0383 |  |  |
| hsa-mir-103b-1 | 5.91 | 1.26 | 0.0070 |  |  |
| hsa-mir-1228 | 5.80 | -1.26 | 0.0383 |  |  |
| hsa-mir-5186 | 5.18 | -1.85 | 0.0070 |  |  |
| hsa-mir-4763 | 5.05 | -2.63 | 0.0003 |  |  |
| hsa-mir-3664 | 4.83 | -1.37 | 0.0454 |  |  |
| hsa-mir-762 | 4.34 | -1.17 | 0.0191 |  |  |
| hsa-mir-1292 | 4.20 | 1.39 | 0.0171 |  |  |
| hsa-mir-520a | 3.79 | -1.47 | 0.0454 |  |  |
| hsa-mir-7848 | 3.69 | -1.92 | 0.0019 |  |  |
| hsa-mir-346 | 3.30 | -1.53 | 0.0118 |  |  |
| hsa-mir-4751 | 2.83 | 1.52 | 0.0454 |  |  |
| hsa-mir-4718 | 2.81 | -1.92 | 0.0328 |  |  |
| hsa-mir-4271 | 2.52 | -1.49 | 0.0383 |  |  |
| hsa-mir-4260 | 2.47 | -1.65 | 0.0136 |  |  |
| hsa-mir-5705 | 2.22 | -2.39 | 0.0361 |  |  |
| hsa-mir-657 | 2.22 | -1.47 | 0.0464 |  |  |
| hsa-mir-8079 | 1.80 | -1.32 | 0.0383 |  |  |
| hsa-mir-3621 | 1.62 | -1.42 | 0.0393 |  |  |
| hsa-mir-564 | 1.58 | -1.60 | 0.0496 |  |  |
| hsa-mir-4651 | 1.50 | -1.56 | 0.0376 |  |  |
| hsa-mir-4322 | 1.49 | -2.25 | 0.0171 |  |  |
| hsa-mir-6858 | 1.45 | -1.95 | 0.0118 |  |  |
| hsa-mir-5000 | 1.30 | -1.82 | 0.0188 |  |  |
| hsa-mir-6878 | 1.29 | -2.36 | 0.0161 |  |  |
| hsa-mir-6722 | 1.17 | -2.16 | 0.0393 |  |  |
| hsa-mir-3978 | 1.14 | -2.28 | 0.0077 |  |  |
| hsa-mir-6857 | 1.10 | -2.09 | 0.0149 |  |  |
| hsa-mir-6731 | 1.04 | -2.28 | 0.0020 |  |  |
| hsa-mir-6774 | 1.01 | -2.54 | 0.0393 |  |  |

Highlighted in yellow: putative NMIBC G1+G2 miRNA biomarkers

In red: common miRNAs between DESeq analyses and Logistic Regression

**B**

| **Differntially Expressed miRNAs** | | | | Regression Model | |
| --- | --- | --- | --- | --- | --- |
| **miRNAs** | Mean read counts | log2 Fold Change | adj p-value (FDR) | **miRNAs** | Prediction power |
| hsa-mir-21 | 38623.72 | 0.87 | 0.03144 | hsa-mir-508 | 0.91 |
| hsa-mir-30a | 29231.61 | -1.67 | 0.00410 | hsa-mir-106b | 0.88 |
| hsa-mir-10b | 28017.52 | -1.88 | 0.00043 | hsa-mir-10b | 0.85 |
| hsa-let-7b | 23816.38 | -0.88 | 0.01635 | hsa-mir-148b | 0.85 |
| hsa-mir-10a | 15925.64 | -1.19 | 0.00314 | hsa-mir-125b-2 | 0.85 |
| hsa-mir-200b | 11969.50 | 0.72 | 0.03357 | hsa-mir-10a | 0.82 |
| hsa-mir-26a-1 | 10968.83 | -0.92 | 0.02029 | hsa-mir-5002 | 0.82 |
| hsa-mir-200c | 9144.98 | 1.11 | 0.00043 | hsa-mir-196a-2 | 0.82 |
| hsa-mir-4448 | 7834.09 | -2.27 | 0.00703 | hsa-mir-6753 | 0.82 |
| hsa-mir-192 | 6415.45 | -1.53 | 0.00539 | hsa-mir-588 | 0.82 |
| hsa-mir-99a | 5068.17 | -1.27 | 0.03144 | hsa-mir-509-1 | 0.82 |
| hsa-mir-200a | 4992.24 | 0.70 | 0.00307 | hsa-mir-8061 | 0.82 |
| hsa-let-7g | 4176.94 | 0.92 | 0.00529 | hsa-mir-98 | 0.79 |
| hsa-mir-151a | 3883.66 | 0.51 | 0.02659 | hsa-let-7e | 0.79 |
| hsa-mir-100 | 3241.94 | -1.46 | 0.00376 | hsa-mir-4459 | 0.79 |
| hsa-let-7c | 2939.10 | -1.17 | 0.02500 | hsa-mir-103a-2 | 0.79 |
| hsa-mir-486 | 2918.41 | 3.14 | 0.00313 | hsa-mir-224 | 0.79 |
| hsa-mir-30c-2 | 2241.38 | -1.47 | 0.01226 | hsa-mir-182 | 0.79 |
| hsa-mir-375 | 2166.27 | -1.53 | 0.02080 | hsa-mir-6800 | 0.79 |
| hsa-mir-532 | 2133.69 | -0.69 | 0.04195 | hsa-mir-4522 | 0.79 |
| hsa-mir-6872 | 2095.56 | 2.08 | 0.00253 | hsa-mir-4800 | 0.79 |
| hsa-mir-451a | 1587.49 | 2.45 | 0.04391 | hsa-mir-206 | 0.79 |
| hsa-mir-3124 | 1490.68 | -2.89 | 0.00245 | hsa-mir-378g | 0.79 |
| hsa-mir-183 | 1413.67 | 1.50 | 0.00094 | hsa-mir-6078 | 0.79 |
| hsa-let-7e | 929.23 | -1.17 | 0.00212 | hsa-mir-548e | 0.79 |
| hsa-mir-204 | 916.77 | -1.97 | 0.00454 | hsa-mir-452 | 0.79 |
| hsa-mir-7641-2 | 795.43 | 1.21 | 0.03795 | hsa-mir-4748 | 0.79 |
| hsa-mir-191 | 769.13 | 0.76 | 0.01490 | hsa-mir-4428 | 0.79 |
| hsa-mir-25 | 734.79 | 2.01 | 0.00008 | hsa-mir-7-1 | 0.76 |
| hsa-mir-205 | 734.31 | 2.06 | 0.00121 | hsa-mir-30a | 0.76 |
| hsa-mir-182 | 675.82 | 0.99 | 0.00058 | hsa-mir-21 | 0.76 |
| hsa-mir-148b | 659.93 | 0.95 | 0.00149 | hsa-let-7g | 0.76 |
| hsa-mir-146b | 527.15 | -1.18 | 0.00349 | hsa-mir-200c | 0.76 |
| hsa-mir-28 | 518.90 | 0.79 | 0.00529 | hsa-mir-934 | 0.76 |
| hsa-mir-224 | 487.60 | 2.97 | 0.00001 | hsa-mir-4792 | 0.76 |
| hsa-mir-98 | 459.11 | 1.01 | 0.01146 | hsa-mir-5003 | 0.76 |
| hsa-mir-222 | 452.65 | -0.90 | 0.02026 | hsa-mir-185 | 0.76 |
| hsa-let-7f-2 | 449.34 | -1.02 | 0.00261 | hsa-mir-3124 | 0.76 |
| hsa-mir-7-1 | 383.11 | 2.21 | 0.00008 | hsa-mir-4430 | 0.76 |
| hsa-mir-185 | 381.47 | 2.10 | 0.00240 | hsa-mir-194-2 | 0.76 |
| hsa-mir-103a-2 | 360.06 | 1.38 | 0.00201 | hsa-mir-127 | 0.76 |
| hsa-mir-194-1 | 329.56 | -1.20 | 0.04684 | hsa-mir-631 | 0.76 |
| hsa-mir-7641-1 | 314.00 | 1.02 | 0.04221 | hsa-mir-485 | 0.76 |
| hsa-mir-9-1 | 211.24 | -1.51 | 0.00775 | hsa-mir-188 | 0.76 |
| hsa-mir-4459 | 207.45 | -1.28 | 0.00306 | hsa-mir-584 | 0.76 |
| hsa-mir-4430 | 192.80 | -1.29 | 0.00775 | hsa-mir-3619 | 0.76 |
| hsa-mir-196a-1 | 185.69 | -1.21 | 0.01898 | hsa-mir-7706 | 0.76 |
| hsa-mir-106b | 183.67 | 2.30 | 0.00000 | hsa-mir-501 | 0.76 |
| hsa-mir-20a | 167.24 | 1.41 | 0.00039 | hsa-mir-1323 | 0.76 |
| hsa-mir-3125 | 163.44 | -3.04 | 0.00044 | hsa-mir-611 | 0.76 |
| hsa-mir-23a | 160.24 | 1.53 | 0.01601 | hsa-mir-199a-2 | 0.76 |
| hsa-mir-93 | 157.62 | 1.89 | 0.00044 | hsa-mir-187 | 0.76 |
| hsa-mir-4792 | 153.45 | 2.31 | 0.00129 | hsa-mir-202 | 0.76 |
| hsa-mir-514a-1 | 144.76 | -2.30 | 0.00493 | hsa-mir-8064 | 0.76 |
| hsa-mir-16-1 | 142.49 | 2.09 | 0.00314 | hsa-mir-324 | 0.76 |
| hsa-mir-629 | 139.92 | 1.17 | 0.03485 | hsa-mir-943 | 0.76 |
| hsa-mir-4497 | 138.47 | 0.95 | 0.01953 | hsa-mir-7703 | 0.76 |
| hsa-mir-210 | 113.42 | 1.83 | 0.00087 | hsa-mir-4744 | 0.76 |
| hsa-mir-516b-2 | 101.06 | 4.40 | 0.00476 | hsa-mir-4804 | 0.76 |
| hsa-mir-95 | 95.91 | -1.15 | 0.04221 | hsa-mir-4750 | 0.76 |
| hsa-mir-584 | 87.43 | 1.42 | 0.00223 | hsa-mir-520f | 0.76 |
| hsa-mir-542 | 87.05 | 0.99 | 0.01445 |  |  |
| hsa-mir-4508 | 85.04 | 0.91 | 0.03144 |  |  |
| hsa-mir-934 | 82.83 | 1.42 | 0.00390 |  |  |
| hsa-mir-4259 | 79.42 | -1.39 | 0.00215 |  |  |
| hsa-mir-4492 | 78.62 | 1.48 | 0.01012 |  |  |
| hsa-mir-425 | 77.89 | 1.90 | 0.00008 |  |  |
| hsa-mir-452 | 68.44 | 2.67 | 0.00009 |  |  |
| hsa-let-7a-3 | 65.80 | -0.97 | 0.02764 |  |  |
| hsa-mir-125b-2 | 65.77 | -1.70 | 0.00233 |  |  |
| hsa-mir-6813 | 65.18 | -2.48 | 0.00002 |  |  |
| hsa-mir-6087 | 63.99 | 1.23 | 0.01713 |  |  |
| hsa-mir-769 | 63.74 | 1.24 | 0.00420 |  |  |
| hsa-mir-1323 | 61.82 | 6.20 | 0.01615 |  |  |
| hsa-mir-6766 | 54.51 | -1.74 | 0.01463 |  |  |
| hsa-mir-144 | 48.34 | 2.57 | 0.00529 |  |  |
| hsa-mir-122 | 46.91 | 1.31 | 0.04319 |  |  |
| hsa-mir-184 | 44.79 | -1.83 | 0.00002 |  |  |
| hsa-mir-517a | 44.58 | 4.83 | 0.04221 |  |  |
| hsa-mir-1911 | 39.13 | -2.33 | 0.00287 |  |  |
| hsa-mir-1301 | 38.94 | 0.90 | 0.03080 |  |  |
| hsa-mir-187 | 36.62 | 1.36 | 0.00206 |  |  |
| hsa-mir-7-3 | 35.86 | -1.86 | 0.00581 |  |  |
| hsa-mir-3180-4 | 34.31 | -2.17 | 0.01814 |  |  |
| hsa-mir-127 | 34.25 | -1.33 | 0.02080 |  |  |
| hsa-mir-548o | 33.53 | -1.86 | 0.00253 |  |  |
| hsa-mir-378c | 32.90 | 0.92 | 0.00990 |  |  |
| hsa-mir-657 | 31.00 | -3.08 | 0.03857 |  |  |
| hsa-mir-330 | 30.20 | 0.80 | 0.02842 |  |  |
| hsa-mir-652 | 30.14 | 1.26 | 0.00480 |  |  |
| hsa-mir-5187 | 28.10 | -4.50 | 0.00129 |  |  |
| hsa-mir-503 | 26.74 | 2.13 | 0.01356 |  |  |
| hsa-mir-196a-2 | 26.62 | -1.36 | 0.01527 |  |  |
| hsa-mir-509-1 | 26.34 | -2.30 | 0.00111 |  |  |
| hsa-mir-4706 | 26.00 | 2.13 | 0.01477 |  |  |
| hsa-mir-8089 | 25.87 | -1.57 | 0.04080 |  |  |
| hsa-mir-1285-2 | 25.40 | -0.94 | 0.02026 |  |  |
| hsa-mir-6883 | 24.44 | 1.24 | 0.02951 |  |  |
| hsa-mir-512-1 | 24.40 | 3.98 | 0.02701 |  |  |
| hsa-mir-133a-2 | 23.97 | -1.58 | 0.01961 |  |  |
| hsa-mir-6800 | 23.73 | -3.68 | 0.00001 |  |  |
| hsa-mir-4634 | 23.36 | 2.18 | 0.00791 |  |  |
| hsa-mir-4327 | 23.31 | -3.05 | 0.00514 |  |  |
| hsa-mir-7706 | 22.46 | 2.22 | 0.00081 |  |  |
| hsa-mir-3945 | 21.82 | -6.71 | 0.03316 |  |  |
| hsa-mir-4657 | 21.71 | -2.66 | 0.00040 |  |  |
| hsa-mir-206 | 20.73 | -2.43 | 0.00565 |  |  |
| hsa-mir-765 | 20.44 | -1.84 | 0.00812 |  |  |
| hsa-mir-497 | 20.27 | -2.06 | 0.00592 |  |  |
| hsa-mir-451b | 19.97 | 3.72 | 0.03163 |  |  |
| hsa-mir-6848 | 19.43 | -1.80 | 0.00123 |  |  |
| hsa-mir-509-3 | 19.33 | -2.63 | 0.00058 |  |  |
| hsa-mir-338 | 18.99 | -1.33 | 0.01473 |  |  |
| hsa-mir-6127 | 18.86 | -1.51 | 0.01126 |  |  |
| hsa-mir-4686 | 17.90 | -3.25 | 0.00687 |  |  |
| hsa-mir-7161 | 17.77 | -2.04 | 0.01580 |  |  |
| hsa-mir-129-1 | 17.55 | -1.79 | 0.01473 |  |  |
| hsa-mir-647 | 17.47 | -2.50 | 0.00253 |  |  |
| hsa-mir-3156-1 | 17.13 | -3.07 | 0.00139 |  |  |
| hsa-mir-105-1 | 14.61 | 3.46 | 0.04199 |  |  |
| hsa-mir-3169 | 14.24 | -2.50 | 0.00407 |  |  |
| hsa-mir-508 | 13.74 | -2.73 | 0.00000 |  |  |
| hsa-mir-616 | 13.56 | -1.59 | 0.00501 |  |  |
| hsa-mir-3160-2 | 13.56 | -2.10 | 0.00514 |  |  |
| hsa-mir-3619 | 13.29 | -2.21 | 0.00307 |  |  |
| hsa-mir-522 | 13.19 | 2.83 | 0.02504 |  |  |
| hsa-mir-3167 | 12.93 | -3.24 | 0.00008 |  |  |
| hsa-mir-558 | 12.31 | -2.04 | 0.00002 |  |  |
| hsa-mir-4674 | 12.30 | -2.48 | 0.00308 |  |  |
| hsa-mir-6080 | 12.09 | -1.63 | 0.01273 |  |  |
| hsa-mir-4537 | 11.43 | -1.99 | 0.01737 |  |  |
| hsa-mir-6845 | 11.27 | -2.58 | 0.00279 |  |  |
| hsa-mir-6068 | 10.99 | -2.17 | 0.01146 |  |  |
| hsa-mir-4687 | 10.90 | -3.35 | 0.03283 |  |  |
| hsa-mir-137 | 10.80 | -2.60 | 0.01741 |  |  |
| hsa-mir-653 | 10.72 | -1.72 | 0.01126 |  |  |
| hsa-mir-324 | 10.71 | 1.17 | 0.02893 |  |  |
| hsa-mir-103b-1 | 10.55 | 1.71 | 0.01728 |  |  |
| hsa-mir-1267 | 10.52 | -4.94 | 0.00514 |  |  |
| hsa-mir-516b-1 | 9.95 | 5.70 | 0.00307 |  |  |
| hsa-mir-5003 | 9.87 | -3.11 | 0.00253 |  |  |
| hsa-mir-663a | 9.86 | 1.21 | 0.04684 |  |  |
| hsa-mir-519a-1 | 9.52 | 3.37 | 0.01079 |  |  |
| hsa-mir-4302 | 9.13 | -2.43 | 0.00034 |  |  |
| hsa-mir-1184-1 | 8.83 | -2.07 | 0.03044 |  |  |
| hsa-mir-4460 | 8.73 | -4.06 | 0.00100 |  |  |
| hsa-mir-1260b | 8.65 | -1.40 | 0.01429 |  |  |
| hsa-mir-432 | 8.58 | -1.93 | 0.02026 |  |  |
| hsa-mir-4647 | 8.45 | -1.79 | 0.00668 |  |  |
| hsa-mir-4740 | 8.44 | -2.52 | 0.00062 |  |  |
| hsa-mir-362 | 8.29 | -1.42 | 0.04255 |  |  |
| hsa-mir-6809 | 8.28 | -2.33 | 0.00413 |  |  |
| hsa-mir-1468 | 8.12 | -1.32 | 0.01728 |  |  |
| hsa-mir-135b | 8.09 | -1.61 | 0.00998 |  |  |
| hsa-mir-548p | 7.93 | -3.25 | 0.00685 |  |  |
| hsa-mir-5002 | 7.92 | -2.28 | 0.00021 |  |  |
| hsa-mir-4800 | 7.86 | -1.92 | 0.00133 |  |  |
| hsa-mir-1253 | 7.48 | -2.10 | 0.01386 |  |  |
| hsa-mir-5698 | 7.42 | -2.08 | 0.01601 |  |  |
| hsa-mir-5708 | 7.33 | -1.45 | 0.04331 |  |  |
| hsa-mir-4443 | 7.15 | -2.84 | 0.01041 |  |  |
| hsa-mir-6753 | 6.99 | -1.95 | 0.00008 |  |  |
| hsa-mir-543 | 6.71 | -1.95 | 0.02504 |  |  |
| hsa-mir-588 | 6.71 | -3.65 | 0.00000 |  |  |
| hsa-mir-5186 | 6.58 | -2.00 | 0.03963 |  |  |
| hsa-mir-548e | 6.36 | -1.36 | 0.02324 |  |  |
| hsa-mir-1228 | 6.36 | -2.14 | 0.01875 |  |  |
| hsa-mir-3972 | 6.33 | -3.27 | 0.00008 |  |  |
| hsa-mir-520g | 6.14 | 3.34 | 0.04803 |  |  |
| hsa-mir-381 | 5.97 | -2.25 | 0.00406 |  |  |
| hsa-mir-4804 | 5.85 | -4.03 | 0.00037 |  |  |
| hsa-mir-611 | 5.79 | -4.25 | 0.00001 |  |  |
| hsa-mir-6832 | 5.76 | -2.92 | 0.00133 |  |  |
| hsa-mir-217 | 5.66 | -2.43 | 0.00349 |  |  |
| hsa-mir-8061 | 5.60 | -3.05 | 0.02510 |  |  |
| hsa-mir-30c-1 | 5.53 | -1.54 | 0.00568 |  |  |
| hsa-mir-6803 | 5.39 | -1.42 | 0.03745 |  |  |
| hsa-mir-1226 | 5.29 | -1.71 | 0.04331 |  |  |
| hsa-mir-4724 | 5.10 | 2.56 | 0.01068 |  |  |
| hsa-mir-147b | 5.08 | 1.79 | 0.03316 |  |  |
| hsa-mir-411 | 5.07 | -2.58 | 0.00389 |  |  |
| hsa-mir-518f | 5.02 | 3.09 | 0.02921 |  |  |
| hsa-mir-766 | 4.82 | -1.48 | 0.03080 |  |  |
| hsa-mir-4306 | 4.69 | -1.56 | 0.04221 |  |  |
| hsa-mir-378d-1 | 4.52 | -2.79 | 0.02764 |  |  |
| hsa-mir-4298 | 4.52 | -2.65 | 0.03237 |  |  |
| hsa-mir-6843 | 4.51 | -2.35 | 0.00133 |  |  |
| hsa-mir-581 | 4.40 | -1.79 | 0.03237 |  |  |
| hsa-mir-1203 | 4.19 | -5.65 | 0.01273 |  |  |
| hsa-mir-3141 | 4.17 | 2.71 | 0.00253 |  |  |
| hsa-mir-1302-5 | 4.05 | -3.64 | 0.00215 |  |  |
| hsa-mir-6867 | 4.00 | -2.24 | 0.04870 |  |  |
| hsa-mir-4748 | 3.96 | -2.26 | 0.00744 |  |  |
| hsa-mir-567 | 3.91 | -1.70 | 0.02026 |  |  |
| hsa-mir-4778 | 3.90 | -2.30 | 0.00349 |  |  |
| hsa-mir-6078 | 3.65 | -1.48 | 0.03257 |  |  |
| hsa-mir-4296 | 3.62 | -1.52 | 0.02552 |  |  |
| hsa-mir-4763 | 3.59 | -1.60 | 0.01867 |  |  |
| hsa-mir-5195 | 3.49 | -2.74 | 0.00237 |  |  |
| hsa-mir-1258 | 3.43 | -2.28 | 0.02573 |  |  |
| hsa-mir-8064 | 3.41 | -2.36 | 0.00775 |  |  |
| hsa-mir-1469 | 3.32 | -2.63 | 0.01214 |  |  |
| hsa-mir-218-2 | 3.31 | -2.87 | 0.00444 |  |  |
| hsa-mir-4522 | 3.20 | -2.85 | 0.00009 |  |  |
| hsa-mir-6793 | 3.20 | -1.74 | 0.01561 |  |  |
| hsa-mir-5093 | 3.18 | -1.96 | 0.04221 |  |  |
| hsa-mir-7845 | 3.03 | -2.02 | 0.01737 |  |  |
| hsa-mir-5739 | 3.03 | -3.69 | 0.00349 |  |  |
| hsa-mir-3915 | 2.96 | -2.50 | 0.02684 |  |  |
| hsa-mir-4323 | 2.96 | -1.81 | 0.02500 |  |  |
| hsa-mir-485 | 2.95 | -2.20 | 0.01429 |  |  |
| hsa-mir-4329 | 2.88 | -2.48 | 0.00308 |  |  |
| hsa-mir-4279 | 2.85 | -1.63 | 0.03237 |  |  |
| hsa-mir-302c | 2.81 | -3.73 | 0.01782 |  |  |
| hsa-mir-2392 | 2.76 | -1.88 | 0.02573 |  |  |
| hsa-mir-3669 | 2.56 | -2.61 | 0.04650 |  |  |
| hsa-mir-520f | 2.50 | 3.64 | 0.00866 |  |  |
| hsa-mir-4269 | 2.41 | -2.36 | 0.02048 |  |  |
| hsa-mir-4651 | 2.41 | -4.17 | 0.01712 |  |  |
| hsa-mir-7703 | 2.41 | -2.62 | 0.00348 |  |  |
| hsa-mir-6756 | 2.39 | -1.72 | 0.03008 |  |  |
| hsa-mir-4311 | 2.35 | -1.78 | 0.01561 |  |  |
| hsa-mir-199a-2 | 2.25 | -2.17 | 0.01279 |  |  |
| hsa-mir-4514 | 2.25 | -4.79 | 0.00245 |  |  |
| hsa-mir-4697 | 2.24 | -2.31 | 0.04221 |  |  |
| hsa-mir-6839 | 2.21 | -2.22 | 0.00543 |  |  |
| hsa-mir-8088 | 2.20 | -3.78 | 0.00359 |  |  |
| hsa-mir-4442 | 2.19 | -1.66 | 0.03008 |  |  |
| hsa-mir-631 | 2.13 | -1.73 | 0.03237 |  |  |
| hsa-mir-608 | 2.12 | -2.53 | 0.03071 |  |  |
| hsa-mir-4307 | 2.10 | -2.42 | 0.04592 |  |  |
| hsa-mir-3122 | 2.08 | -4.74 | 0.00810 |  |  |
| hsa-mir-5007 | 2.00 | -3.20 | 0.03163 |  |  |
| hsa-mir-4428 | 1.94 | -2.52 | 0.00253 |  |  |
| hsa-mir-4744 | 1.90 | -2.21 | 0.04080 |  |  |
| hsa-mir-3689d-1 | 1.89 | -2.56 | 0.01574 |  |  |
| hsa-mir-1289-2 | 1.88 | -3.47 | 0.00786 |  |  |
| hsa-mir-4788 | 1.85 | -3.33 | 0.00539 |  |  |
| hsa-mir-4712 | 1.78 | -3.17 | 0.00520 |  |  |
| hsa-mir-5192 | 1.71 | -2.91 | 0.04221 |  |  |
| hsa-mir-4252 | 1.66 | -1.95 | 0.02764 |  |  |
| hsa-mir-6729 | 1.65 | -3.19 | 0.00937 |  |  |
| hsa-mir-202 | 1.58 | -2.03 | 0.01192 |  |  |
| hsa-mir-6794 | 1.53 | -4.51 | 0.01796 |  |  |
| hsa-mir-422a | 1.50 | -1.95 | 0.01881 |  |  |
| hsa-mir-1537 | 1.42 | -3.91 | 0.00775 |  |  |
| hsa-mir-4689 | 1.40 | -2.65 | 0.02610 |  |  |
| hsa-mir-4280 | 1.37 | -2.74 | 0.02842 |  |  |
| hsa-mir-3183 | 1.33 | -2.06 | 0.03008 |  |  |
| hsa-mir-7107 | 1.32 | -2.32 | 0.04145 |  |  |
| hsa-mir-4282 | 1.21 | -4.67 | 0.00514 |  |  |
| hsa-mir-4756 | 1.21 | -2.79 | 0.03144 |  |  |
| hsa-mir-5088 | 1.20 | -2.19 | 0.04868 |  |  |
| hsa-mir-3621 | 1.17 | -2.15 | 0.04221 |  |  |
| hsa-mir-6831 | 1.09 | -3.32 | 0.01015 |  |  |
| hsa-mir-4798 | 1.06 | -3.13 | 0.01760 |  |  |
| hsa-mir-4486 | 1.05 | -2.71 | 0.02258 |  |  |
| hsa-mir-4469 | 1.02 | -2.56 | 0.02610 |  |  |
| hsa-mir-6825 | 0.85 | -2.84 | 0.04569 |  |  |
| hsa-mir-3662 | 0.85 | -3.31 | 0.01881 |  |  |
| hsa-mir-4790 | 0.80 | -3.65 | NA |  |  |
| hsa-mir-4761 | 0.58 | -2.92 | NA |  |  |
| hsa-mir-4736 | 0.47 | -4.40 | NA |  |  |

Highlighted in yellow: putative NMIBC G3 miRNA biomarkers

In red: common miRNAs between DESeq analyses and Logistic Regression

**C**

| **Differntially Expressed miRNAs** | | | | Regression Model | |
| --- | --- | --- | --- | --- | --- |
| **miRNAs** | Mean read counts | log2 Fold Change | adj p-value (FDR) | **miRNAs** | Prediction power |
| hsa-mir-205 | 375.80 | 2.97 | 0.0003 | hsa-mir-4706 | 0.90 |
| hsa-mir-106b | 130.01 | 2.53 | 0.0016 | hsa-mir-205 | 0.90 |
| hsa-mir-21 | 29222.96 | 1.42 | 0.0123 | hsa-mir-10b | 0.85 |
| hsa-mir-486 | 819.00 | 3.68 | 0.0144 | hsa-mir-7-1 | 0.85 |
| hsa-mir-25 | 588.45 | 1.99 | 0.0195 | hsa-mir-30d | 0.85 |
| hsa-let-7c | 1496.77 | -1.62 | 0.0250 | hsa-mir-30a | 0.85 |
| hsa-mir-451a | 1004.00 | 3.40 | 0.0400 | hsa-mir-21 | 0.85 |
| hsa-mir-146a | 419.83 | 2.49 | 0.0400 | hsa-mir-99a | 0.85 |
| hsa-mir-7-1 | 518.24 | 3.32 | 0.0467 | hsa-let-7c | 0.85 |
| hsa-mir-30a | 19452.92 | -1.80 | 0.0481 | hsa-mir-6073 | 0.85 |
| hsa-mir-4706 | 34.73 | 4.60 | 0.0003 | hsa-mir-27b | 0.85 |
| hsa-mir-483 | 23.73 | 5.78 | 0.0032 | hsa-mir-24-1 | 0.85 |
| hsa-mir-4792 | 157.88 | 3.06 | 0.0059 | hsa-mir-103a-2 | 0.85 |
| hsa-mir-210 | 76.03 | 1.60 | 0.0141 | hsa-mir-125b-2 | 0.85 |
| hsa-mir-185 | 260.84 | 1.89 | 0.0152 | hsa-mir-9-1 | 0.85 |
| hsa-mir-8089 | 20.02 | -1.87 | 0.0162 | hsa-mir-106b | 0.85 |
| hsa-mir-93 | 172.90 | 2.29 | 0.0219 | hsa-mir-17 | 0.85 |
| hsa-mir-125b-2 | 43.29 | -1.94 | 0.0250 | hsa-mir-3615 | 0.85 |
| hsa-mir-183 | 915.48 | 1.83 | 0.0252 | hsa-mir-18a | 0.85 |
| hsa-mir-206 | 12.65 | -2.34 | 0.0252 | hsa-mir-8089 | 0.85 |
| hsa-mir-23a | 239.00 | 2.91 | 0.0259 | hsa-mir-1269a | 0.85 |
| hsa-mir-125b-1 | 174.16 | -1.71 | 0.0259 | hsa-mir-320e | 0.85 |
| hsa-mir-7854 | 15.41 | 4.09 | 0.0299 | hsa-mir-548h-2 | 0.85 |
| hsa-mir-584 | 46.00 | 1.71 | 0.0400 | hsa-mir-5703 | 0.85 |
| hsa-mir-1302-2 | 34.63 | -5.20 | 0.0400 | hsa-mir-2116 | 0.85 |
| hsa-mir-4473 | 8.02 | 3.77 | 0.0400 | hsa-mir-100 | 0.80 |
| hsa-mir-6829 | 6.08 | -2.44 | 0.0400 | hsa-mir-744 | 0.80 |
| hsa-mir-182 | 10458.88 | 2.45 | 0.0481 | hsa-mir-200c | 0.80 |
| hsa-mir-1290 | 36.24 | 2.57 | 0.0481 | hsa-mir-210 | 0.80 |
| hsa-mir-3928 | 7.09 | 4.02 | 0.0481 | hsa-let-7e | 0.80 |
| hsa-mir-122 | 85.26 | 2.46 | 0.0487 | hsa-mir-184 | 0.80 |
| hsa-mir-6087 | 47.44 | 1.87 | 0.0487 | hsa-mir-941-1 | 0.80 |
| hsa-mir-98 | 241.95 | 1.31 | 0.0499 | hsa-mir-182 | 0.80 |
|  |  |  |  | hsa-mir-4430 | 0.80 |
|  |  |  |  | hsa-mir-20a | 0.80 |
|  |  |  |  | hsa-mir-1827 | 0.80 |
|  |  |  |  | hsa-mir-653 | 0.80 |
|  |  |  |  | hsa-mir-92a-2 | 0.80 |
|  |  |  |  | hsa-mir-206 | 0.80 |
|  |  |  |  | hsa-mir-193b | 0.80 |
|  |  |  |  | hsa-mir-425 | 0.80 |
|  |  |  |  | hsa-mir-93 | 0.80 |
|  |  |  |  | hsa-mir-330 | 0.80 |
|  |  |  |  | hsa-mir-7706 | 0.80 |
|  |  |  |  | hsa-mir-891a | 0.80 |
|  |  |  |  | hsa-mir-937 | 0.80 |
|  |  |  |  | hsa-mir-3180-1 | 0.80 |
|  |  |  |  | hsa-mir-6782 | 0.80 |

Highlighted in yellow: putative MIBC miRNA biomarkers

In red: common miRNAs between DESeq analyses and Logistic Regression
